# Refining Single-Atom Catalytic Kinetics for Tumor Homologous-Targeted Catalytic Therapy

**DOI:** 10.1007/s40820-025-01735-y

**Published:** 2025-05-12

**Authors:** Hengke Liu, Shan Lei, Hongyu Li, Jiayingzi Wu, Ting He, Jing Lin, Peng Huang

**Affiliations:** Marshall Laboratory of Biomedical Engineering, Guangdong Key Laboratory for Biomedical Measurements and Ultrasound Imaging, Laboratory of Evolutionary Theranostics (LET), International Cancer Center, School of Biomedical Engineering, Shenzhen University Medical School, Shenzhen University, Shenzhen, 518055 People’s Republic of China

**Keywords:** Single-atom nanozymes, Glucose oxidase, Biomimetic cascade catalysis, Tumor catalytic therapy, Mild-temperature photothermal therapy

## Abstract

Single-atom nanozymes (SAzymes) hold significant potential for tumor catalytic therapy, but their effectiveness is often compromised by low catalytic efficiency within tumor microenvironment. This efficiency is mainly influenced by key factors including hydrogen peroxide (H_2_O_2_) availability, acidity, and temperature. Simultaneous optimization of these key factors presents a significant challenge for tumor catalytic therapy. In this study, we developed a comprehensive strategy to refine single-atom catalytic kinetics for enhancing tumor catalytic therapy through dual-enzyme-driven cascade reactions. Iridium (Ir) SAzymes with high catalytic activity and natural enzyme glucose oxidase (GOx) were utilized to construct the cascade reaction system. GOx was loaded by Ir SAzymes due to its large surface area. Then, the dual-enzyme-driven cascade reaction system was modified by cancer cell membranes for improving biocompatibility and achieving tumor homologous targeting ability. GOx catalysis reaction could produce abundant H_2_O_2_ and lower the local pH, thereby optimizing key reaction-limiting factors. Additionally, upon laser irradiation, Ir SAzymes could raise local temperature, further enhancing the catalytic efficiency of dual-enzyme system. This comprehensive optimization maximized the performance of Ir SAzymes, significantly improving the efficiency of catalytic therapy. Our findings present a strategy of refining single-atom catalytic kinetics for tumor homologous-targeted catalytic therapy.

**Supplementary Information:**

The online version contains supplementary material available at 10.1007/s40820-025-01735-y.

## Highlights

- Developed a dual-enzyme cascade system integrating iridium single-atom nanozymes with glucose oxidase, optimizing hydrogen peroxide production, acidity, and temperature within the tumor microenvironment to markedly improve catalytic therapy efficacy.
- Functionalized the cascade reaction system with cancer cell membranes to achieve homologous targeting and high biocompatibility, ensuring accurate accumulation at tumor sites while minimizing off-target effects.

**Supplementary Information:**

The online version contains supplementary material available at 10.1007/s40820-025-01735-y.

## Graphical abstract

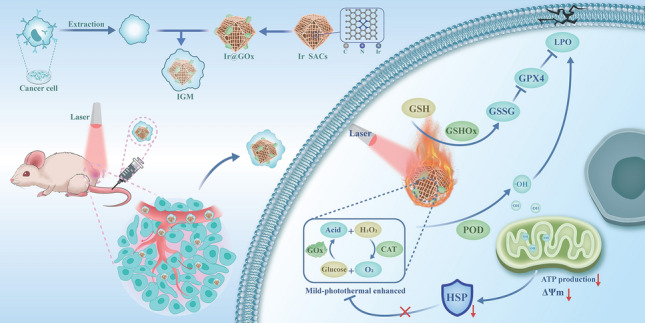

## Introduction

Tumor catalytic therapy, especially single-atom nanozymes (SAzymes)-mediated approaches, has emerged a research hotspot in cancer treatment due to its ability to specifically generate reactive oxygen species (ROS) in tumor tissues [1–3]. The development of atomic nanotechnology has ushered in the era of SAzymes, which provide superior catalytic efficiency and customizable active sites, marking a great advancement over traditional nanoparticle-based catalysts [4, 5]. Recently, highly active SAzymes with well-defined metal–nitrogen–carbon (M–N–C, M = Mn, Fe, Co, Cu, Zn, Ru, etc.) coordination structures have been developed [6–11]. Notably, iridium (Ir) SAzyme demonstrates good biocompatibility and catalytic stability, making them promising candidates for tumor catalytic therapy [12, 13]. SAzyme-based catalytic therapy primarily focuses on regulating ROS level. Various strategies of improving SAzyme performance have been explored to enhance ROS yield, such as defect engineering [14, 15], doping with heterogeneous atoms [13], constructing near-neighbor monometallic atoms [16–19], or coupling with carriers [20–23]. The effectiveness of SAzyme-based therapies depends on both their catalytic activity and their adaptability to variations in the intratumoral microenvironment. Catalytic rates are influenced by factors such as temperature, pH, and hydrogen peroxide (H_2_O_2_) levels within tumor tissues [24–27]. Therefore, optimizing these parameters of catalytic process is crucial for efficient catalytic therapy.

In addition to optimizing catalytic kinetics, the development of an efficient delivery system is equally crucial. Current delivery systems for SAzyme encounter limitations, including low targeting efficiency, insufficient tumor accumulation and short blood circulation time. Recently, cancer cell membranes, as emerging biomimetic carriers, show great potential in the development of efficient delivery systems [28–30]. Cancer cell membrane-camouflaged nanoparticles retain key adhesion proteins, antigens, and membrane structures, preserving the surface characteristics and functions of the source cells [31, 32]. This approach enables homologous targeting, improved biocompatibility, extended blood circulation time, and enhanced cellular uptake compared to synthetic alternatives [28, 33]. Despite the significance of optimizing cascade reaction kinetics and enhancing tumor targeting, there have been limited studies exploring SAzyme-based cascade nanoreactors that can achieve high catalytic activity, sustained H_2_O_2_ supply, reduced pH level, and heat generation—all of which are vital for effective tumor catalytic therapy.

Herein, we develop a strategy to comprehensively optimize the catalytic kinetics of SAzymes for traceable, multistage-enhanced catalytic therapy using a biomimetic cascade nanoreactor (Ir SAzymes@GOx@4T1M, IGM). The IGM was constructed by the combination of Ir SAzyme and natural enzyme glucose oxidase (GOx), followed by cancer cell membrane camouflage. The product H_2_O_2_ generated by GOx catalytic reaction can be used as the substrate of Ir SAzymes. Ir SAzymes-catalyzed reaction was optimized by producing H_2_O_2_ and reducing pH level through glucose oxidation reaction [34, 35]. Moreover, locally moderate heating further enhances the catalytic activity of both Ir SAzymes and GOx [36–39], yielding more cytotoxic hydroxyl radicals (**·**OH). Meanwhile, Ir SAzymes also exhibit catalase (CAT)-like activity, breaking down H_2_O_2_ into O_2_, thus supporting the aerobic catalysis of GOx. Interestingly, the IGM exhibited high tumor accumulation through homotypic targeting of cell membrane camouflage. Ultimately, we comprehensively optimized the catalytic kinetics of Ir SAzymes and synchronously combined GOx, thus achieving dual-enzyme-driven cascade reactions and efficient catalytic therapy.

## Experimental Section

### Materials

Ir(acac)_3_, Zn(NO_3_)_2_·6H_2_O, and glucose oxidase were obtained from Sigma-Aldrich (USA). 3,3',5,5'-tetramethylbenzidine (TMB) were purchased from Shanghai Macklin Biochemical Co., Ltd. 2′,7′-Dichlorofluorescein diacetate (DCFH-DA), calcein-AM, propidium iodide (PI), enhanced BCA protein assay kit, Cell Counting Kit-8 (CCK-8), apoptosis detection kit (Annexin V-FITC/PI) and mitochondrial membrane potential assay kit with JC-1 were purchased from Beyotime Institute of Biotechnology (China). All the chemicals and solvents were used without further purification. Deionized water (18.2 MΩ cm) was used throughout the study.

### Characterizations

Transmission electron microscopy (TEM) images were obtained using an HT7700 transmission electron microscope (Hitachi Electronics, accelerating voltage 80 kV). Elemental mapping analysis was performed using a scanning transmission electron microscopy (JEM-3200FS, Tokyo, Japan) with an accelerating voltage of 300 kV. High-angle annular dark-field scanning transmission electron microscopy (HAADF-STEM) characterization was performed using a double spherical aberration-corrected transmission electron microscope (Titan Cubed Themis G2 300, Thermo Fisher, USA). X-ray powder diffraction (XRD) pattern was recorded in the 2θ range from 10° to 70° using a D8 Advance diffractometer (Bruker, Germany). The X-ray photoelectron spectroscopy (XPS) analysis was performed using ESCALAB 250Xi (Thermo Fisher, USA). Fourier-transform infrared (FT-IR) spectra were collected by an FT-IR spectrometer (Spectrum Two, PerkinElmer, USA) with the wavenumber range from 4000 to 400 cm^–1^ at 4 cm^–1^ resolution and 32 scans per sample. Zeta potential was measured using Zetasizer Nano-ZS90 (Malvern, England). The contents of Ir elements were measured using an inductively coupled plasma instrument (ICP-OES, Optima 7000DV, PerkinElmer, USA). UV/vis absorption spectra were measured using Cary 60 UV/vis spectrophotometer (Agilent Technologies, Santa Clara, CA, USA). Photoacoustic imaging images were obtained using anVevo LAZR-X (VisualSonics Inc. New York, NY).

### Synthesis of Ir SAzymes@GOx@4T1M (IGM)

Ir SAzymes was thoroughly ground and dispersed in water by sonication, and GOx was added to the Ir SAzymes solution at a 1:10 mass ratio of GOx to Ir SAzymes and stirred overnight. Free GOx was removed by centrifugation, and the resulting precipitates were collected to obtain Ir SAzymes@GOx. Next, 1 mg Ir SAzyme@GOx was mixed with an equal volume of 1 mg mL^−1^ (protein concentration) cell membrane vesicles in deionized water. The mixture was sonicated for 10 min using a bath sonicator, followed by centrifugation (10,000 rpm, 10 min) to collect Ir SAzymes@GOx@4T1M (IGM) which was then stored at 4 °C for use. The cell membrane coating was then characterized by SDS-PAGE and TEM.

## Results and Discussion

### Preparation and Characterization of Ir SAzymes

**Fig. 1:**
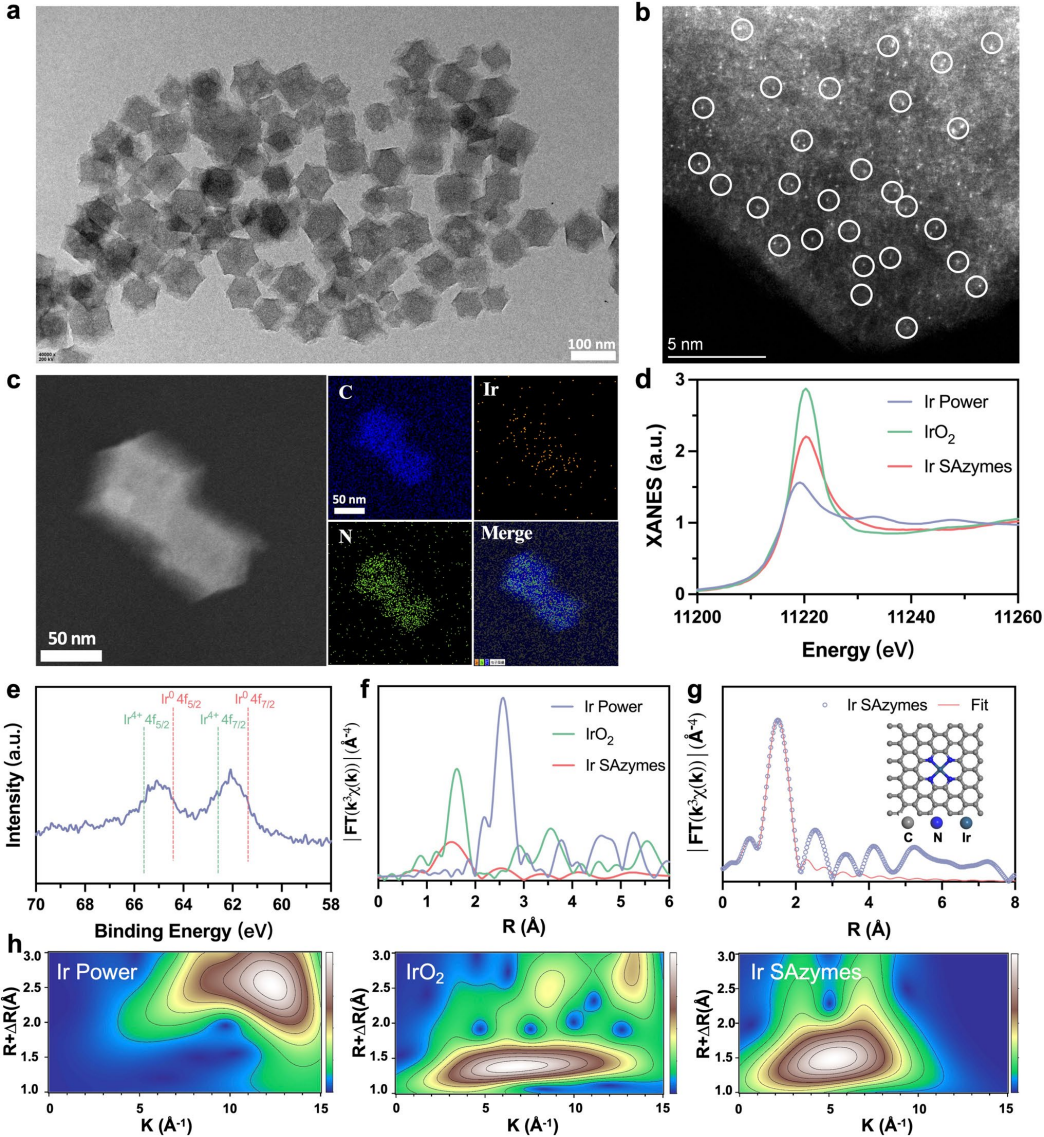
Characterization of Ir SAzymes. **a** HRTEM, **b** Magnified HAADF-STEM image of Ir SAzymes. **c** Element mapping images of Ir SAzymes. **d** XANES of Ir powder, IrO_2_, and Ir SAzymes at the Ir L_3_-edge. **e** XPS spectra of Ir SAzymes for Ir 4*f* regions. **f** FT-EXAFS spectra of Ir powder, IrO_2_ and Ir SAzymes at the iridium L_3_-edge. **g** The corresponding R-space EXAFS fitting curves of Ir SAzymes. The inset illustrates the schematic model of the Ir SAzymes. **h** Wavelet transform plot of Ir L_3_-edge EXAFS of Ir powder, IrO_2_, and Ir SAzymes

Ir SAzymes were synthesized by the pyrolysis of Ir(acac)_3_–loaded ZIF-8, resulting in a structure of single Ir atom dispersed in nitrogen-doped carbon skeleton. During the pyrolysis process, the ZIF-8 framework prevented Ir atom aggregation. TEM and scanning electron microscopy (SEM) images revealed the rhombododecahedral structure of Ir(acac)_3_@ZIF-8 nanocrystals (Fig. S1). High-resolution TEM (HRTEM) and high-angle annular dark-field scanning TEM (HAADF-STEM) images confirmed the absence of large Ir nanoparticles, while the distribution of single Ir atom was observed, as indicated by white circles (Fig. 1a, b). Element mapping images showed the uniform distribution of Ir, C, and N (Fig. 1c). Inductively coupled plasma atomic emission spectrometry (ICP-OES) quantified –0.39 wt% Ir content in Ir SAzymes. As shown in X-ray diffraction (XRD) pattern, the broad peaks were assigned to amorphous graphitic carbon, without crystalline peaks of metallic Ir (Fig. S2). X-ray photoelectron spectroscopy (XPS) spectra identified pyrrolic N, pyridinic N, and graphitic N as anchoring sites for Ir atoms, ensuring single-atom dispersion (Fig. S3). X-ray absorption near-edge structure (XANES) analysis displayed the coordination environment of Ir atoms and showed absorption energies between metallic Ir and IrO_2_, suggesting partially positive Ir atoms (Fig. 1d), which is consistent with Ir 4*f* XPS spectrum (Fig. 1e). Fourier-transformed extended X-ray absorption fine structure (FT-EXAFS) curves showed a peak at –1.6 Å, indicating Ir-N coordination, with no Ir-Ir peak around 2.6 Å, confirming isolated single Ir atom (Fig. 1f). EXAFS fitting curves indicated an average Ir-N bond length of 1.98 Å (Table S1). Density functional theory (DFT) calculation identified most stable configuration as single Ir atom coordinated with four N atoms (Fig. 1g). Wavelet transform (WT) analysis further supported the FT-EXAFS results, showing maximum intensity at 5.1 Å^−1^ for Ir-N coordination (Fig. 1h). Compared to WT plots of Ir powder and IrO_2_, no Ir-Ir intensity was observed. These results confirmed the successful synthesis of Ir SAzymes.

### Photothermal Property and Multienzyme Activities of Ir SAzymes

**Fig. 2:**
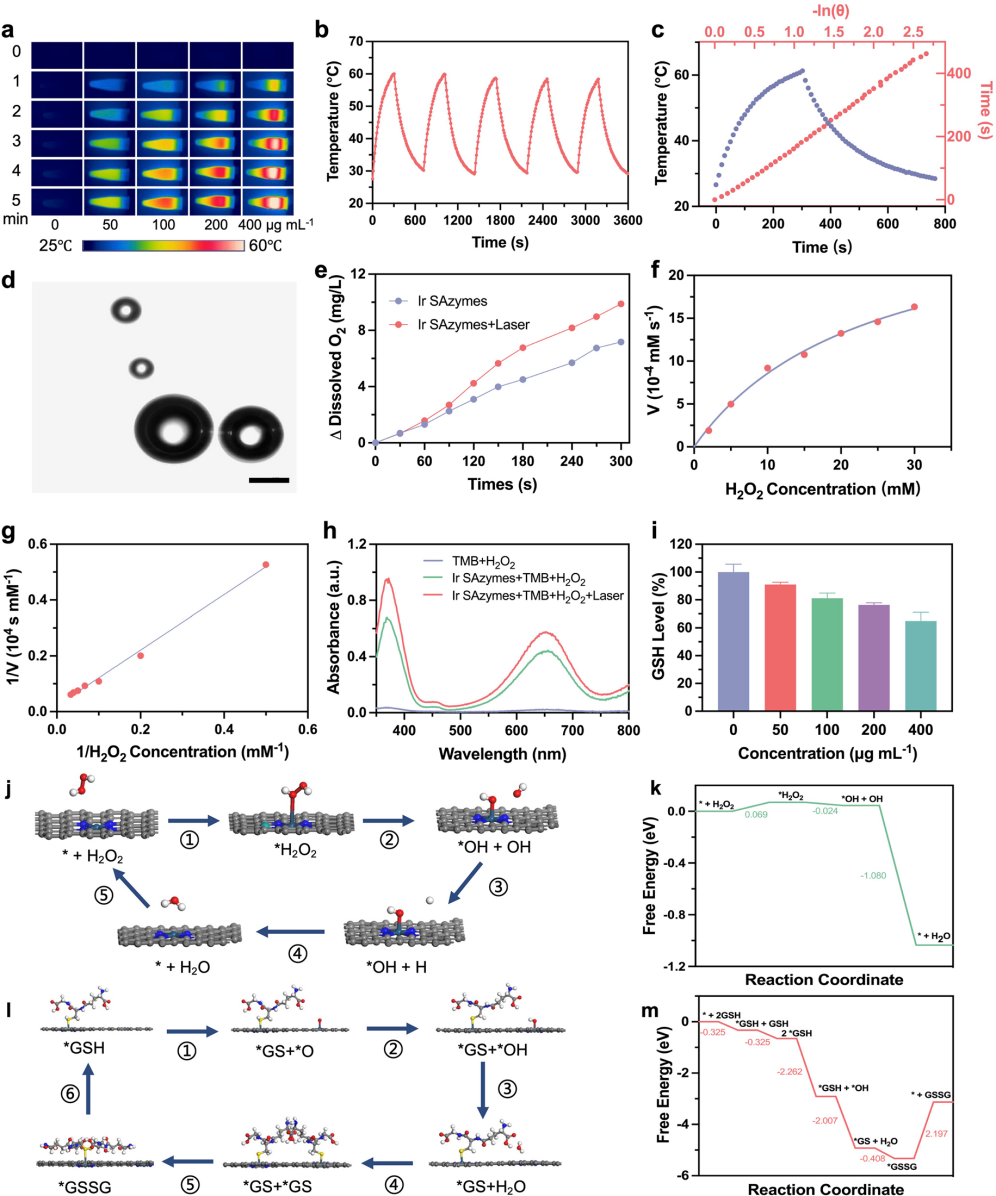
**a** Thermal images of Ir SAzymes aqueous solutions under laser irradiation (808 nm, 0.8 W cm^−2^). **b** Photothermal stability of Ir SAzymes (400 μg mL^−1^) under five successive laser on/off cycles. **c** PCE of Ir SAzymes. **d** Confocal microscopy images of O_2_ bubbles in an Ir SAzymes and H_2_O_2_ mixture. Scale bar: 200 μm. **e** O_2_ generation in various groups (pH = 6.5). Ir SAzymes concentration: 50 μg mL^−1^, H_2_O_2_ concentration: 1 mM. **f** Kinetic analysis of Ir SAzymes with POD-like activity. The concentration of H_2_O_2_ was varied in the presence of TMB (500 μM). **g** Double-reciprocal plots of Ir SAzymes activities derived from the Michaelis–Menten equation. **h** UV–Vis absorption spectra of TMB solution with and without 808 nm laser irradiation (0.8 W cm^−2^, 3 min) **i** GSH depletion after incubation with different concentrations of Ir SAzymes. **j** Schematic illustration of the catalytic mechanism and **k** corresponding free energy diagrams of POD-like activity of Ir SAzymes. **l** Schematic illustration of the catalytic mechanism and **m** corresponding free energy diagrams of GSHOx-like activity of Ir SAzymes. Cyan, blue, gray, red, white, and yellow spheres represent Ir, N, C, O, H, and S atoms, respectively

The Brunauer–Emmett–Teller (BET) measurement indicated that the Ir SAzymes exhibited a surface area of 821.3 m^2^ g⁻^1^ and an average pore diameter of 8.9 nm, suggesting that the large surface area and porous structure benefit the exposure of single Ir active site and GOx loading (Figs. S4 and S5). The GOx loading capacity was calculated to be 1.7 wt%, as determined by the bicinchoninic acid (BCA) protein assay. According to the Arrhenius equation, the reaction rate constant increases exponentially with temperature, remarkably enhancing the catalytic reaction rate [40, 41]. The ultraviolet–visible-near infrared (UV–Vis-NIR) spectrum of Ir SAzymes revealed strong absorption in NIR region with an extinction coefficient of 5.46 L g⁻^1^ cm⁻^1^ at 808 nm (Fig. S6). Upon 808 nm laser irradiation, the temperature of the Ir SAzymes dispersion markedly increased in a concentration-dependent manner (Figs. 2a and S7). Ir SAzymes exhibited good photothermal stability and a photothermal conversion efficiency (PCE) of 38.1% (Fig. 2b, c).

The multienzyme mimetic activities of Ir SAzymes were further investigated. The CAT-like activity of Ir SAzymes in H_2_O_2_ decomposition was observed using confocal laser scanning microscopy (CLSM), visualizing the generation of O_2_ bubbles. Experiments quantifying H_2_O_2_ consumption showed oxygen-generating capability of Ir SAzymes, especially under laser irradiation, indicating that the CAT-like activity could be enhanced by photothermal effect (Fig. 2d, e). The decomposition of H_2_O_2_ by Ir SAzymes follows by Michaelis–Menten kinetics equation with a K_m_ value of 49.41 mM (Fig. S8), which is equivalent to natural CAT (52.14 mM) [42]. A colorimetric reaction using 3,3',5,5'-tetramethylbenzidine dihydrochloride (TMB) followed Michaelis–Menten kinetics, with a Menten constant (K_m_) of 23.74 mM and a maximum reaction rate (V_max_) of 2.89 μM s⁻^1^ (Figs. 2f, g and S9). Furthermore, laser irradiation increased TMB oxidation by 1.34-fold compared to the non-irradiated group (Fig. 2h). These findings indicated that laser irradiation enhanced both CAT-like and POD-like activities. According to the Arrhenius equation, the reaction rate constant increased exponentially with temperature, thus accelerating the catalytic rate. Therefore, the observed enhancement of enzyme activity is attributed to the photothermal effect. As shown in Fig. 2i, the decreased absorption intensity of 5-thio-2-nitrobenzoic acid (TNB) at 412 nm indicated the effective glutathione oxidase (GSHOx)-like activity of Ir SAzymes, which could further enhance tumor catalytic therapy.

To understand the catalytic mechanism and structure–activity relationship of Ir SAzymes, DFT calculations were performed. For POD-like activity, the H_2_O_2_ molecule initially adsorbs onto single Ir atoms on Ir SAzymes with an adsorption energy of 0.069 eV (Fig. 2j, k). The dissociation of activated H_2_O_2_ could generate a reactive ·OH and an attached hydroxyl group (*OH) by the Ir site. Subsequently, the attached *OH reacted with a protonated hydrogen atom to produce H_2_O. This process was accompanied by an obvious Gibbs free energy decrease (-1.08 eV). Hence, DFT analysis revealed that Ir SAzymes exhibited POD-like activity. For GSHOx-like activity, GSH molecule spontaneously adsorbed onto Ir atom and could be dissociated in the presence of O and OH groups from H_2_O to form *GS. The generated *GS subsequently coupled to form GSSG, the total Gibbs free energy decrease (-3.13 eV), indicating high GSHOx-like activity of Ir SAzymes (Fig. 2l, m).

### Construction of the Dual-Enzyme-Driven Cascade Reaction Platform

**Fig. 3:**
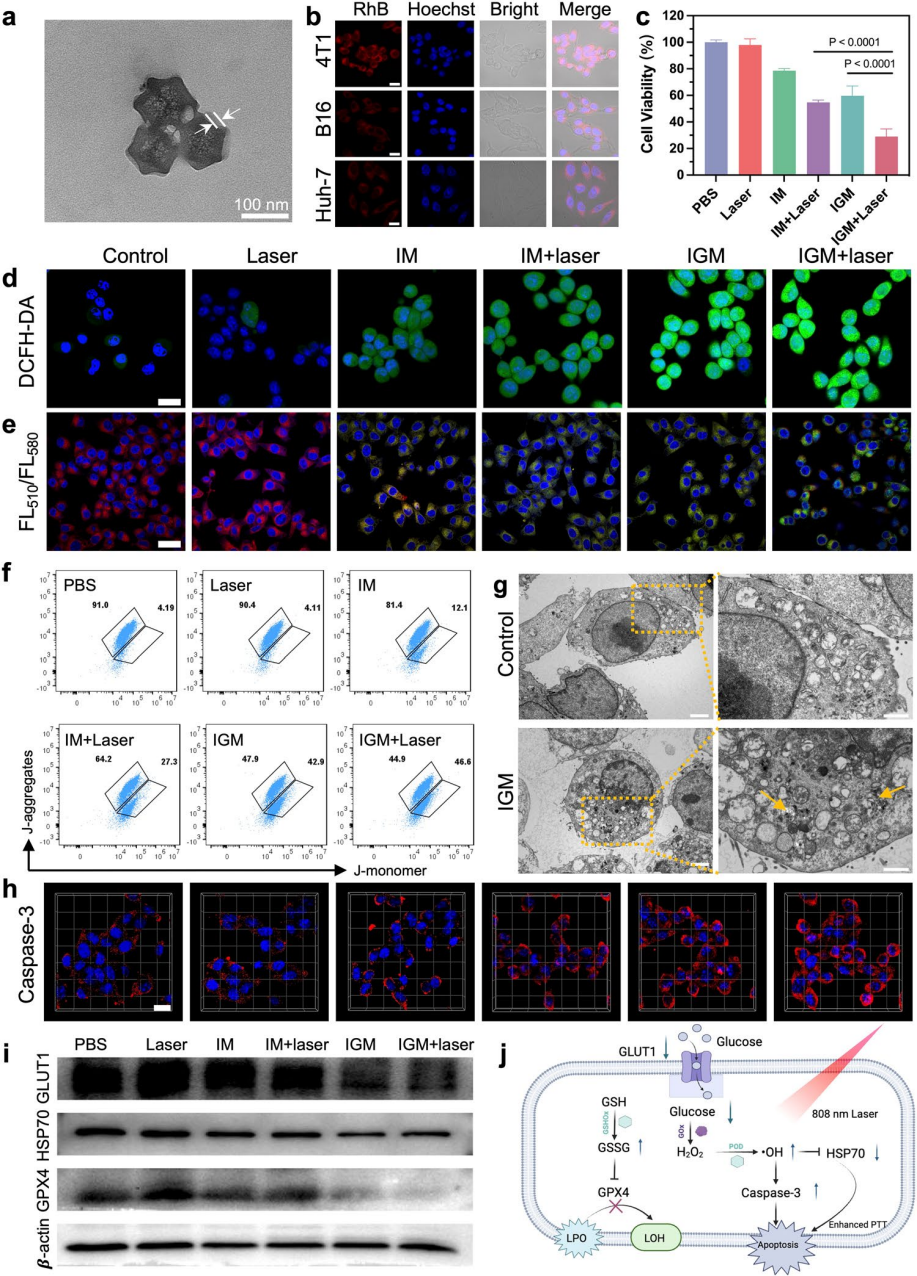
**a** TEM images of the synthesized IGM. **b** Fluorescence images of various cells treated with RhB-labeled IGM, Scale bar: 20 μm. **c** Cytotoxicity assessment of 4T1 cells with different treatments. **d** Confocal images of 4T1 cells stained with DCFH-DA, and **e** C11-BODIPY^581/591^ after various treatments. Scale bar: 20 μm.** f** Flow cytometry analysis of the J-monomer and J-aggregates after various treatments. **g** Bio-TEM images of 4T1 cells with different treatments (orange arrow indicate the IGM; scale bars: 2 μm and 1 μm, respectively). **h** Confocal images of 4T1 cells stained with activation of caspase-3 after various treatments. Scale bar: 20 μm. **i** Western blots of GLUT1, HSP70, and GPX4 after various treatments. **j** Potential antitumor mechanism of IGM plus laser-mediated catalytic therapy

To improve the biocompatibility and homologous targeting ability, cancer cell membranes nanovesicles (Fig. S10) derived from 4T1 cells were used to encapsulate Ir SAzymes through hydrophobic interactions [32, 43], producing cancer cell membrane-cloaked Ir SAzyme@GOx (IGM). TEM images showed a thick coating layer of 4T1 cell membrane surrounding the rhombododecahedron-shaped Ir SAzymes (Fig. 3a). The FT-IR analysis revealed that the composition of IGM is characterized by strong peaks corresponding to both GOx (1656 cm⁻^1^, indicating C = O group) and 4T1M (1096 cm⁻^1^, corresponding to the C-O-P group), confirming the formation of IGM composites (Fig. S11). After coating, the zeta potential of Ir SAzymes changed from 5.6 ± 1.1 to -14.3 ± 4.2 mV (Fig. S12). SDS-PAGE analysis further confirmed the successful modification of 4T1 cell membranes (Fig. S13). DLS analysis indicated that the hydrodynamic size of Ir SAzymes was 196.6 nm, increasing to 226.3 nm after cell membrane coating, suggesting successful surface modification (Fig. S14a). In addition, IGM exhibited no obvious size change over seven days in various physiological solutions, verifying its good stability (Fig. S14b).

Encouraged by the high catalytic performance of SAzymes, in vitro anticancer activity of IGM was further assessed. The cytotoxicity of Ir SAzymes@Membrane (IM) was assessed against MCF-10A (normal cells) and 4T1 (tumor cells) using a Cell Counting Kit-8 (CCK-8) assay. The findings indicated negligible cytotoxic effects on MCF-10A cells, even at high concentrations, while 4T1 cell viability was significantly reduced. This suggests that IM was selectively activated in an acidic environment (Fig. S15). Efficient uptake of SAzymes by tumor cells is crucial for effective tumor therapy. Flow cytometry showed rapid IGM uptake by 4T1 cells with time-dependent fluorescence enhancement (Fig. S16). To verify homologous targeting, 4T1, B16, and Huh-7 cells were incubated with Rhodamine B (RhB)-labeled IGM nanoparticles (RhB-IGM) and examined with CLSM (Fig. 3b, S17). The red fluorescence intensity in 4T1 cells was 2.4- and 2.7-fold higher than that of in B16 and Huh-7 cells, respectively, demonstrating its homologous targeting capability [28]. After verifying the effective cellular internalization provided by the 4T1 cell membrane camouflage, next, the in vitro cascade catalytic efficacy of IGM was evaluated. After treatment with IGM (40 μg mL^−1^), 4T1 cell viability decreased by 40.2%, whereas IM alone exhibited mild toxicity (21.3%) (Fig. 3c). Upon 808 nm laser irradiation for 5 min, IGM treatment exhibited obvious cytotoxicity, suggesting the dual-enzyme-driven cascade reaction could be enhanced by photothermal effect. Flow cytometry analysis with Annexin V and propidium iodide (PI) co-staining also confirmed the highest apoptosis rate in the IGM plus laser group (Figs. S18 and S19).

### In Vitro Enhanced Catalytic Therapy of IGM

To further elucidate the mechanism of cell death, we studied the variation of intracellular molecular events associated with the cascade reaction. The obvious fluorescence signal of 2′,7′-dichlorofluorescein diacetate (DCFH-DA) in IGM treatment group showed that the IGM could induce ROS generation, which was further enhanced by laser irradiation (Figs. 3d and S20). ROS accumulation plays a pivotal role in lipid peroxidation (LPO) of cell membranes and DNA damage, ultimately inducing cell death [44, 45]. LPO was evaluated using the ratiometric fluorescent probe C11-BODIPY^581/591^. The IGM + Laser treatment group exhibited a strong green fluorescence signal and a weak red signal, indicating enhanced ROS generation and subsequent LPO under laser irradiation (Fig. 3e). Alterations in mitochondrial membrane potential (MMP) were assessed using the JC-1 fluorescent probe and showed that the ratio of JC-1 monomers (low MMP) in the IGM-treated group (42.9%) was 3.54-fold higher than that of in the IM-treated group (12.1%), suggesting the mitochondrial depolarization by cascade catalysis (Fig. 3f). Additionally, IGM treatment resulted in the lower adenosine triphosphate (ATP) levels in tumor cells, suggesting mitochondrial dysfunction (Fig. S21). The ROS-related cytotoxicity of IGM was further confirmed through bio-TEM imaging. Reduced cell size and nuclear chromatin condensation, hallmarks of apoptosis, were observed in cells treated with IGM (Fig. 3g). Upon laser irradiation, IGM induced significant cell apoptosis, as demonstrated by the elevated expression of caspase-3 (Figs. 3h and S22). Subsequently, the combined therapeutic molecular mechanism was investigated. Glucose transporter 1 (GLUT1) is a glucose transporter protein that is upregulated and promotes tumor cell growth and survival [46]. Notably, GLUT1 expression was down-regulated in cells treated with IGM, and further reduced by laser irradiation (Figs. 3i and S23). IGM treatment led to obvious down-regulation of HSP70, suggesting ROS inhibition of heat shock proteins (HSPs) [47, 48]. The IGM treatment showed LPO accumulation and reduced glutathione peroxidase 4 (GPX4) expression, illustrating GPX4 inactivation along with GSH consumption. Figure 3j summarizes the potential molecular mechanisms underlying IGM-mediated catalytic therapy. Our study revealed that the combination of IGM and laser irradiation effectively reduces ATP levels, increases ·OH levels, downregulates GLUT1, GPX4, and HSP70, and activates caspase-3, thereby efficiently promoting cell apoptosis. These results suggest that optimizing the catalytic kinetics of the dual-enzyme system involves multiple molecular events within live cells, coupled with feedback mechanisms that synergistically enhanced tumor treatment efficacy.

### In Vitro and In Vivo Imaging

**Fig. 4:**
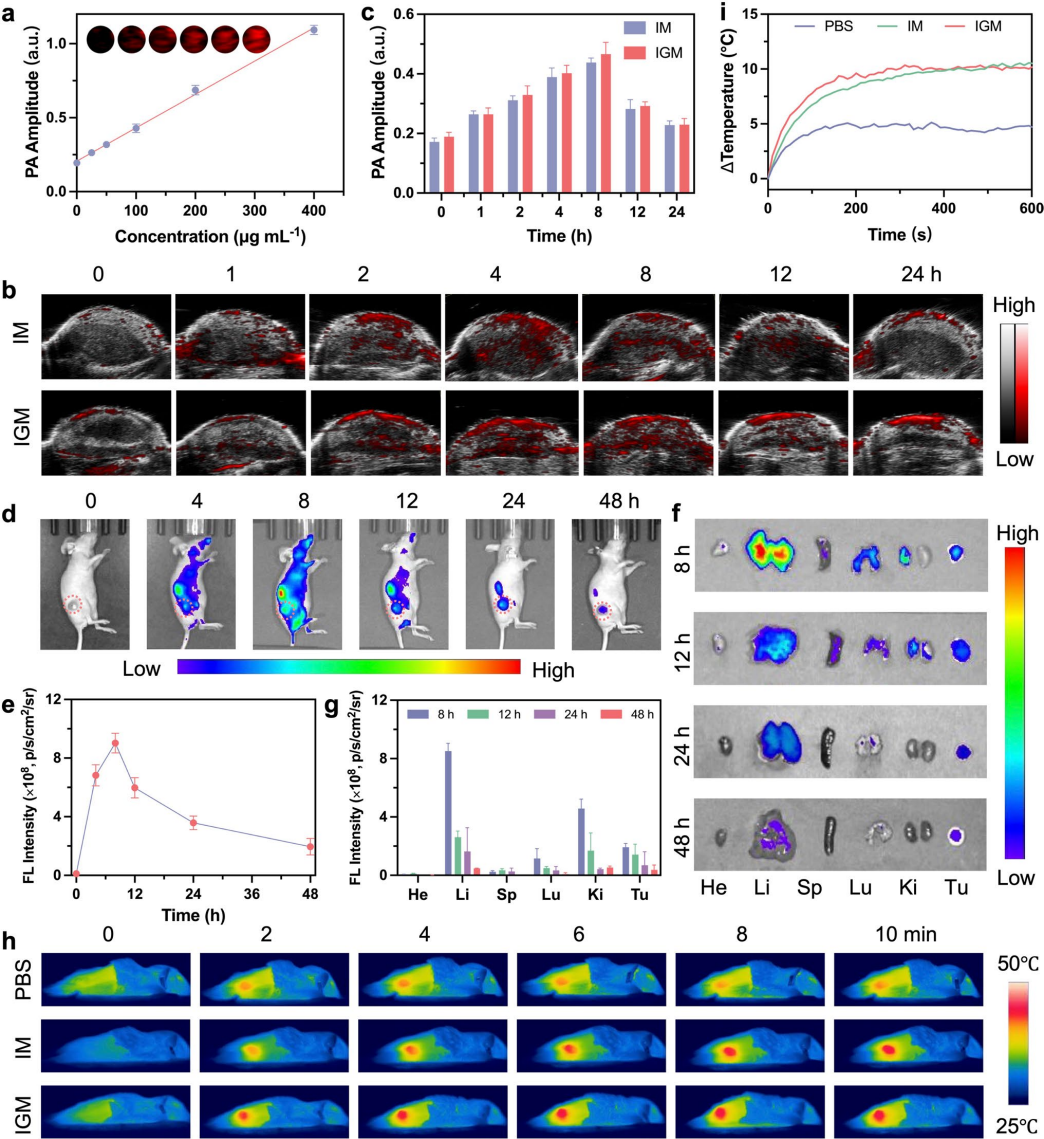
**a** PA images and corresponding PA values at various IGM solution concentrations under 808 nm laser irradiation. **b** In vivo PA images and **c** quantification of PA amplitudes at tumor sites after *i.v.* injection of IM and IGM, presented as mean ± SD (n = 3). **d** Representative fluorescence images and **e** corresponding fluorescence intensity of tumor in mice at various time points after *i.v.* injection of IGM. **f** Representative fluorescence images of dissected organs and tumors at 8, 12, 24, and 48 h after injection of IGM-IR800, and **g** corresponding fluorescence intensity of major organs and tumor tissues. Data are presented as mean ± SD (n = 3). **h** Thermal images of mice after *i.v.* injection of PBS, IM, and IGM (808 nm, 0.8 W cm⁻^2^, 10 min) and **i** corresponding changes in tumor temperature

Inspired by the promising in vitro therapeutic effects of IGM, we conducted its antitumor effects in vivo. Initially, the accumulation of IGM at the tumor site was investigated to determine the optimal time window for laser-enhanced catalytic therapy. As a powerful imaging technique, photoacoustic (PA) imaging noninvasively and precisely traces the distribution of accumulated nanoparticles at the tumor site with high resolution [49, 50]. Given the photothermal properties of Ir SAzymes under laser irradiation, we evaluated the PA imaging potential of IGM both in vitro and in vivo. As shown in Fig. 4a, a clear PA signal proportional to the concentration of IGM was observed, suggesting that IGM could act as a PA contrast agent. For in vivo PA imaging, the IGM was administered via intravenous injection into 4T1 tumor-bearing mice. The PA signal intensity increased approximately 2.47-fold at 8-h post-injection compared to the initial signal at 0 h (Fig. 4b, c), suggesting that IGM could efficiently accumulate at the tumor site through homologous targeting. To track the metabolic behavior of IGM, we labeled IGM with a near infrared dye, IRdye 800CW NHS ester (IR800), for in vivo fluorescence imaging. Following intravenous injection of IGM-IR800, the fluorescence intensity of the tumor reached its peak at 8 h and remained elevated up to 48 h. This prolonged signal indicates that the cancer cell membrane camouflage effectively maintained high enrichment of IGM in the tumor tissue (Fig. 4d, e). Additionally, mice were sacrificed at various time points (8, 12, 24, and 48 h), and major organs and tumors were harvested to quantify the distribution of IGM (Fig. 4f, g). Fluorescence images of major organs revealed that IGM was primarily metabolized by the liver and kidneys. Furthermore, to investigate the pharmacokinetic profile of IGM in 4T1 tumor-bearing mice, blood samples were collected at different time intervals post-injection to assess drug concentrations. The blood half-life of IGM was found to be 4.14 ± 1.56 h (Fig. S24, Table S2), which contributes to improved tumor accumulation and therapeutic potential. Next, we evaluated the in vivo therapeutic efficacy of IGM in a 4T1 tumor-bearing mouse model. We firstly performed in vivo photothermal imaging to assess the generation of localized hyperthermia. Compared to the PBS group, IGM-treated mice exhibited a rapid temperature increase within the tumor region under 808 nm laser irradiation, with the maximum temperature reaching 45.2 °C within 10 min (Fig. 4h, i).

### In Vivo Enhanced Catalytic Therapy of IGM

**Fig. 5:**
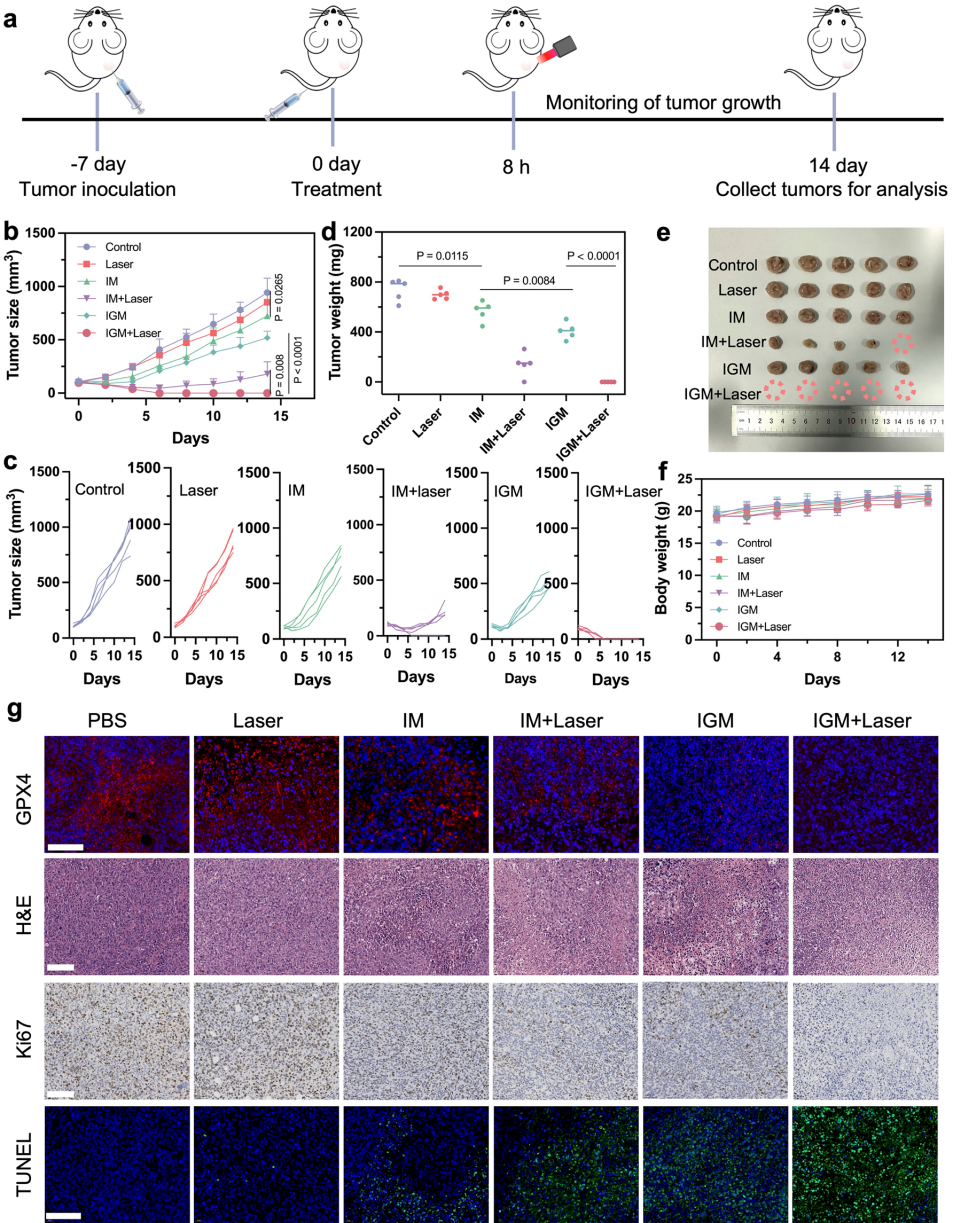
**a** Treatment protocol for the antitumor experiment on 4T1 tumor-bearing mice. **b** Tumor growth curves after the indicated treatments, presented as mean ± SD (n = 5). **c** Individual tumor growth curves for each group after various treatments. **d** Tumor weight of mice after the indicated treatments. **e** Photographs of xenograft tumors excised from mice after 14 days of treatment. **f** Average body weight of mice throughout the indicated treatments. **g** GPX4, H&E, Ki67, and TUNEL staining images of tumor sections following various treatments. Scale bar: 100 μm

After that, the in vivo antitumor effect of IGM was evaluated in 4T1 tumor-bearing mice. The 4T1 tumor-bearing mice were randomly divided into six groups (n = 5 per group): Control (PBS), Laser, IM, IM + Laser, IGM, and IGM + Laser. Detailed methodologies for the construction of the mouse model and subsequent interventions are presented in Fig. 5a. Tumor growth curves showed that PBS or laser treatment alone exhibited negligible inhibition effect on tumors. In contrast, IGM showed superior catalytic therapeutic efficacy compared to IM, which was further enhanced by laser irradiation, underscoring the robust in vivo cascade catalytic therapeutic efficacy of IGM (Fig. 5b, c). Individual tumor growth curves and photographs of excised tumors showed complete tumor clearance in the IGM + Laser group, demonstrating the effectiveness of the fully optimized single-atom catalytic therapy (Fig. 5d, e). During the treatment period, all mouse groups showed a slight increase in body weight (Fig. 5f). Additionally, immunofluorescence imaging revealed a significant reduction in GPX4 and HSP70 expression levels, attributed to the IGM-induced ferroptosis response (Figs. 5g, S25 and S26). To further investigate the antitumor efficacy of IGM, tumor sections from different groups were stained with hematoxylin and eosin (H&E), Ki-67, and TdT-mediated dUTP nick-end labeling (TUNEL) for histological analysis (Figs. 5g and S27). The IGM + Laser group showed the most significant cell damage and the highest apoptotic cell population, underscoring the strong therapeutic potential of this optimized single-atom catalytic therapy in vivo. Importantly, hemolysis and blood biochemistry analysis (Figs. S28 and S29) demonstrated the good biocompatibility of IGM. Meanwhile, the H&E staining images of major organs showed no inflammation or obvious tissue damage (Fig. S30), collectively confirming the good biocompatibility of IGM for in vivo theranostic applications. Collectively, these experimental results indicated that IGM exhibited effective antitumor effect and good biocompatibility, offering a promising strategy to enhance single-atom nanozyme-mediated catalytic therapy.

## Conclusion

In summary, we developed an optimized strategy to enhance single-atom catalytic kinetics for traceable, multistage catalytic therapy using a biomimetic cascade nanoreactor (IGM). By leveraging cancer cell membrane camouflage, IGM effectively targeted tumors, with substantial tumor accumulation confirmed through in vivo PA imaging. GOx-assisted catalysis intensified the intratumoral catalytic process of Ir SAzymes by further acidifying the tumor microenvironment and increasing H_2_O_2_ availability, thereby facilitating ·OH radical generation. By further enhancing the catalytic rate of dual-enzyme system through laser irradiation, IGM show good therapeutic potential both in vitro and in vivo. This study addresses the key limitations of tumor catalytic therapy, including inadequate tumor targeting, low catalytic efficiency, and insufficient H_2_O_2_ availability within the tumor microenvironment. Consequently, this strategy offers a promising pathway to enhance intratumoral catalytic kinetics and improve the efficacy of SAzymes-mediated tumor catalytic therapy.

## Supplementary Information

Below is the link to the electronic supplementary material.

Supplementary file1 (DOCX 5712 KB)

## Declarations

### Supporting Information

Some details of cell and animal experiments, additional results including XRD pattern, Cell uptake, photographs of the experimental process, H&E staining images of normal tissues, and other additional information.

## References

[1] S. Lei, J. Zhang, N.T. Blum, M. Li, D.-Y. Zhang et al., *In vivo* three-dimensional multispectral photoacoustic imaging of dual enzyme-driven cyclic cascade reaction for tumor catalytic therapy. Nat. Commun. **13**(1), 1298 (2022). 10.1038/s41467-022-29082-1 35277519 10.1038/s41467-022-29082-1 PMC8917194

[2] H. Lin, Y. Chen, J. Shi, Nanoparticle-triggered *in situ* catalytic chemical reactions for tumour-specific therapy. Chem. Soc. Rev. **47**(6), 1938–1958 (2018). 10.1039/c7cs00471k 29417106 10.1039/c7cs00471k

[3] H. Xiang, W. Feng, Y. Chen, Single-atom catalysts in catalytic biomedicine. Adv. Mater. **32**(8), e1905994 (2020). 10.1002/adma.201905994 31930751 10.1002/adma.201905994

[4] H. Zhang, X.F. Lu, Z.P. Wu, X.W.D. Lou, Emerging multifunctional single-atom catalysts/nanozymes. ACS Cent. Sci. **6**(8), 1288–1301 (2020). 10.1021/acscentsci.0c00512 32875072 10.1021/acscentsci.0c00512 PMC7453415

[5] Y. Liu, H. Zhao, Y. Zhao, Designing efficient single metal atom biocatalysts at the atomic structure level. Angew. Chem. Int. Ed. **63**(13), e202315933 (2024). 10.1002/anie.202315933 10.1002/anie.202315933 38206594

[6] S. Cai, J. Liu, J. Ding, Z. Fu, H. Li et al., Tumor-microenvironment-responsive cascade reactions by a cobalt-single-atom nanozyme for synergistic nanocatalytic chemotherapy. Angew. Chem. Int. Ed. **61**(48), e202204502 (2022). 10.1002/anie.202204502 10.1002/anie.202204502 35972794

[7] Y. Zhu, W. Wang, J. Cheng, Y. Qu, Y. Dai et al., Stimuli-responsive manganese single-atom nanozyme for tumor therapy *via* integrated cascade reactions. Angew. Chem. Int. Ed. **60**(17), 9480–9488 (2021). 10.1002/anie.202017152 10.1002/anie.202017152 33543825

[8] S. Ji, B. Jiang, H. Hao, Y. Chen, J. Dong et al., Matching the kinetics of natural enzymes with a single-atom iron nanozyme. Nat. Catal. **4**(5), 407–417 (2021). 10.1038/s41929-021-00609-x

[9] Z. Zhao, H. Tan, P. Zhang, X. Liang, T. Li et al., Turning the inert element zinc into an active single-atom catalyst for efficient Fenton-like chemistry. Angew. Chem. Int. Ed. **62**(18), e202219178 (2023). 10.1002/anie.202219178 10.1002/anie.202219178 36892151

[10] D. Wang, J. Wang, X.J. Gao, H. Ding, M. Yang et al., Employing noble metal-porphyrins to engineer robust and highly active single-atom nanozymes for targeted catalytic therapy in nasopharyngeal carcinoma. Adv. Mater. **36**(7), e2310033 (2024). 10.1002/adma.202310033 37994246 10.1002/adma.202310033

[11] M. Chang, Z. Hou, M. Wang, D. Wen, C. Li et al., Cu single atom nanozyme based high-efficiency mild photothermal therapy through cellular metabolic regulation. Angew. Chem. Int. Ed. **61**(50), e202209245 (2022). 10.1002/anie.202209245 10.1002/anie.202209245 36264713

[12] Z. Wang, W. Wang, J. Wang, D. Wang, M. Liu et al., Single-atom catalysts with ultrahigh catalase-like activity through electron filling and orbital energy regulation. Adv. Funct. Mater. **33**(2), 2209560 (2023). 10.1002/adfm.202209560

[13] Y. Liu, B. Wang, J. Zhu, X. Xu, B. Zhou et al., Single-atom nanozyme with asymmetric electron distribution for tumor catalytic therapy by disrupting tumor redox and energy metabolism homeostasis. Adv. Mater. **35**(9), e2208512 (2023). 10.1002/adma.202208512 36373624 10.1002/adma.202208512

[14] B. Yu, W. Wang, W. Sun, C. Jiang, L. Lu, Defect engineering enables synergistic action of enzyme-mimicking active centers for high-efficiency tumor therapy. J. Am. Chem. Soc. **143**(23), 8855–8865 (2021). 10.1021/jacs.1c03510 34086444 10.1021/jacs.1c03510

[15] J. Liu, B. Yu, M. Rong, W. Sun, L. Lu, A new strategy to fight tumor heterogeneity: Integrating metal-defect active centers within NADH oxidase nanozymes. Nano Today **54**, 102113 (2024). 10.1016/j.nantod.2023.102113

[16] Y. Liu, R. Niu, R. Deng, S. Song, Y. Wang et al., Multi-enzyme co-expressed dual-atom nanozymes induce cascade immunogenic ferroptosis *via* activating interferon-γ and targeting arachidonic acid metabolism. J. Am. Chem. Soc. **145**(16), 8965–8978 (2023). 10.1021/jacs.2c13689 37058189 10.1021/jacs.2c13689

[17] R. Zeng, Q. Gao, L. Xiao, W. Wang, Y. Gu et al., Precise tuning of the D-band center of dual-atomic enzymes for catalytic therapy. J. Am. Chem. Soc. **146**(14), 10023–10031 (2024). 10.1021/jacs.4c00791 38554097 10.1021/jacs.4c00791

[18] R. Zeng, Y. Li, X. Hu, W. Wang, Y. Li et al., Atomically site synergistic effects of dual-atom nanozyme enhances peroxidase-like properties. Nano Lett. **23**(13), 6073–6080 (2023). 10.1021/acs.nanolett.3c01454 37358396 10.1021/acs.nanolett.3c01454

[19] G. Li, H. Liu, T. Hu, F. Pu, J. Ren et al., Dimensionality engineering of single-atom nanozyme for efficient peroxidase-mimicking. J. Am. Chem. Soc. **145**(30), 16835–16842 (2023). 10.1021/jacs.3c05162 37487021 10.1021/jacs.3c05162

[20] J. Yang, H. Yao, Y. Guo, B. Yang, J. Shi, Enhancing tumor catalytic therapy by co-catalysis. Angew. Chem. Int. Ed. **61**(17), e202200480 (2022). 10.1002/anie.202200480 10.1002/anie.202200480 35143118

[21] G. Feng, H. Huang, M. Zhang, Z. Wu, D. Sun et al., Single atom iron-doped graphic-phase C_3_N_4_ semiconductor nanosheets for augmented sonodynamic melanoma therapy synergy with endowed chemodynamic effect. Adv. Sci. **10**(23), e2302579 (2023). 10.1002/advs.202302579 10.1002/advs.202302579 PMC10427360 37282773

[22] J. Zhou, D. Xu, G. Tian, Q. He, X. Zhang et al., Coordination-driven self-assembly strategy-activated Cu single-atom nanozymes for catalytic tumor-specific therapy. J. Am. Chem. Soc. **145**(7), 4279–4293 (2023). 10.1021/jacs.2c13597 10.1021/jacs.2c13597 36744911

[23] H. Yang, M. Luo, S. Lu, Q. Zhang, Y. Chao et al., Low-temperature aerobic oxidation of thiophenic sulfides over atomic Mo hosted by cobalt hydroxide sub-nanometer sheets. Chem **8**(9), 2460–2471 (2022). 10.1016/j.chempr.2022.06.001

[24] W. He, J. Wu, J. Liu, J. Li, Single-atom nanozymes for catalytic therapy: recent advances and challenges. Adv. Funct. Mater. **34**(16), 2312116 (2024). 10.1002/adfm.202312116

[25] C. Peng, R. Pang, J. Li, E. Wang, Current advances on the single-atom nanozyme and its bioapplications. Adv. Mater. **36**(10), e2211724 (2024). 10.1002/adma.202211724 36773312 10.1002/adma.202211724

[26] J. Liu, A. Wang, S. Liu, R. Yang, L. Wang et al., A titanium nitride nanozyme for pH-responsive and irradiation-enhanced cascade-catalytic tumor therapy. Angew. Chem. Int. Ed. **60**(48), 25328–25338 (2021). 10.1002/anie.202106750 10.1002/anie.202106750 34453387

[27] L. Yang, S. Dong, S. Gai, D. Yang, H. Ding et al., Deep insight of design, mechanism, and cancer theranostic strategy of nanozymes. Nano-Micro Lett. **16**(1), 28 (2023). 10.1007/s40820-023-01224-0 10.1007/s40820-023-01224-0 PMC10663430 37989794

[28] J. Cui, F. Zhang, D. Yan, T. Han, L. Wang et al., “Trojan horse” phototheranostics: fine-engineering NIR-II AIEgen camouflaged by cancer cell membrane for homologous-targeting multimodal imaging-guided phototherapy. Adv. Mater. **35**(33), 2302639 (2023). 10.1002/adma.202302639 10.1002/adma.202302639 37161639

[29] B. Geng, J. Hu, Y. Li, S. Feng, D. Pan et al., Near-infrared phosphorescent carbon dots for sonodynamic precision tumor therapy. Nat. Commun. **13**(1), 5735 (2022). 10.1038/s41467-022-33474-8 36175446 10.1038/s41467-022-33474-8 PMC9523047

[30] Z. Liu, L. Zhang, T. Cui, M. Ma, J. Ren et al., A nature-inspired metal-organic framework discriminator for differential diagnosis of cancer cell subtypes. Angew. Chem. Int. Ed. **60**(28), 15436–15444 (2021). 10.1002/anie.202102286 10.1002/anie.202102286 33960090

[31] M. Liu, Y. Feng, Y. Lu, R. Huang, Y. Zhang et al., Lymph-targeted high-density lipoprotein-mimetic nanovaccine for multi-antigenic personalized cancer immunotherapy. Sci. Adv. **10**(11), eadk2444 (2024). 10.1126/sciadv.adk2444 10.1126/sciadv.adk2444 PMC10936870 38478602

[32] R.H. Fang, W. Gao, L. Zhang, Targeting drugs to tumours using cell membrane-coated nanoparticles. Nat. Rev. Clin. Oncol. **20**(1), 33–48 (2023). 10.1038/s41571-022-00699-x 36307534 10.1038/s41571-022-00699-x

[33] Y. Zou, Y. Sun, Y. Wang, D. Zhang, H. Yang et al., Cancer cell-mitochondria hybrid membrane coated Gboxin loaded nanomedicines for glioblastoma treatment. Nat. Commun. **14**(1), 4557 (2023). 10.1038/s41467-023-40280-3 37507371 10.1038/s41467-023-40280-3 PMC10382535

[34] L.-H. Fu, C. Qi, J. Lin, P. Huang, Catalytic chemistry of glucose oxidase in cancer diagnosis and treatment. Chem. Soc. Rev. **47**(17), 6454–6472 (2018). 10.1039/c7cs00891k 30024579 10.1039/c7cs00891k

[35] L.H. Fu, C. Qi, Y.R. Hu, J. Lin, P. Huang, Glucose oxidase-instructed multimodal synergistic cancer therapy. Adv. Mater. **31**(21), 1808325 (2019). 10.1002/adma.201808325 10.1002/adma.201808325 30907460

[36] J. Wu, Y. Zhang, K. Jiang, X. Wang, N.T. Blum et al., Enzyme-engineered conjugated polymer nanoplatform for activatable companion diagnostics and multistage augmented synergistic therapy. Adv. Mater. **34**(18), e2200062 (2022). 10.1002/adma.202200062 35243699 10.1002/adma.202200062

[37] T. He, H. Xu, Y. Zhang, S. Yi, R. Cui et al., Glucose oxidase-instructed traceable self-oxygenation/hyperthermia dually enhanced cancer starvation therapy. Theranostics **10**(4), 1544–1554 (2020). 10.7150/thno.40439 32042321 10.7150/thno.40439 PMC6993236

[38] C. Yang, M.R. Younis, J. Zhang, J. Qu, J. Lin et al., Programmable NIR-II photothermal-enhanced starvation-primed chemodynamic therapy using glucose oxidase-functionalized ancient pigment nanosheets. Small **16**(25), e2001518 (2020). 10.1002/smll.202001518 32468633 10.1002/smll.202001518

[39] X. Zhang, X. Chen, Y. Zhao, Nanozymes: versatile platforms for cancer diagnosis and therapy. Nano-Micro Lett. **14**(1), 95 (2022). 10.1007/s40820-022-00828-2 10.1007/s40820-022-00828-2 PMC8986955 35384520

[40] J. Kohout, Modified Arrhenius equation in materials science, chemistry and biology. Molecules **26**(23), 7162 (2021). 10.3390/molecules26237162 34885745 10.3390/molecules26237162 PMC8658926

[41] M. Zandieh, J.W. Liu, Nanozymes: Definition, activity, and mechanisms. Adv. Mater. **36**, 2211041 (2024). 10.1002/adma.202211041 10.1002/adma.202211041 36799556

[42] R. Zhang, B. Xue, Y. Tao, H. Zhao, Z. Zhang et al., Edge-site engineering of defective Fe-N_4_ nanozymes with boosted catalase-like performance for retinal vasculopathies. Adv. Mater. **34**(39), e2205324 (2022). 10.1002/adma.202205324 35953446 10.1002/adma.202205324

[43] J. Cheng, L. Li, D. Jin, Y. Dai, Y. Zhu et al., Boosting ferroptosis therapy with iridium single-atom nanocatalyst in ultralow metal content. Adv. Mater. **35**(17), e2210037 (2023). 10.1002/adma.202210037 36718883 10.1002/adma.202210037

[44] Q. Hu, W. Zhu, J. Du, H. Ge, J. Zheng et al., A GPX4-targeted photosensitizer to reverse hypoxia-induced inhibition of ferroptosis for non-small cell lung cancer therapy. Chem. Sci. **14**(34), 9095–9100 (2023). 10.1039/d3sc01597a 37655031 10.1039/d3sc01597a PMC10466276

[45] J. Liu, S. Dong, S. Gai, Y. Dong, B. Liu et al., Design and mechanism insight of monodispersed AuCuPt alloy nanozyme with antitumor activity. ACS Nano **17**(20), 20402–20423 (2023). 10.1021/acsnano.3c06833 37811650 10.1021/acsnano.3c06833

[46] D. Deng, C. Xu, P. Sun, J. Wu, C. Yan et al., Crystal structure of the human glucose transporter GLUT1. Nature **510**(7503), 121–125 (2014). 10.1038/nature13306 24847886 10.1038/nature13306

[47] F. Zeng, L. Tang, Q. Zhang, C. Shi, Z. Huang et al., Coordinating the mechanisms of action of ferroptosis and the photothermal effect for cancer theranostics. Angew. Chem. Int. Ed. **61**(13), e202112925 (2022). 10.1002/anie.202112925 10.1002/anie.202112925 34932846

[48] K. Li, K. Xu, S. Liu, Y. He, M. Tan et al., All-in-one engineering multifunctional nanoplatforms for sensitizing tumor low-temperature photothermal therapy *in vivo*. ACS Nano **17**(20), 20218–20236 (2023). 10.1021/acsnano.3c05991 37838975 10.1021/acsnano.3c05991

[49] B. Xu, S. Li, L. Zheng, Y. Liu, A. Han et al., A bioinspired five-coordinated single-atom iron nanozyme for tumor catalytic therapy. Adv. Mater. **34**(15), e2107088 (2022). 10.1002/adma.202107088 35102632 10.1002/adma.202107088

[50] L. Liu, Y. Wu, J. Ye, Q. Fu, L. Su et al., Synthesis of magnesium nanoparticle for NIR-II-photoacoustic-imaging-guided synergistic burst-like and H_2_ cancer therapy. Chem **8**(11), 2990–3007 (2022). 10.1016/j.chempr.2022.07.001

