# Supplementary material for: Refining Single-Atom Catalytic Kinetics for Tumor Homologous-Targeted Catalytic Therapy
Source: Nanomicro Lett. 2025 May 12;17:253. doi: 10.1007/s40820-025-01735-y (PMC12069810; doi:10.1007/s40820-025-01735-y)
Supplement: Supplementary file 1 — Supplementary file1 (DOCX 5712 KB) [file 40820_2025_1735_MOESM1_ESM.docx]

Supporting Information for

Refining Single-Atom Catalytic Kinetics for Tumor Homologous-Targeted Catalytic Therapy

Hengke Liu^1,‡^, Shan Lei^1,‡^, Hongyu Li^1,‡^, Jiayingzi Wu^1^, Ting He^1^, Jing Lin^1^, and Peng Huang^1^*

^1^Marshall Laboratory of Biomedical Engineering, International Cancer Center, Guangdong Key Laboratory for Biomedical Measurements and Ultrasound Imaging, Laboratory of Evolutionary Theranostics (LET), School of Biomedical Engineering, Shenzhen University Medical School, Shenzhen University, Shenzhen, 518055, P. R. China

‡Hengke Liu, Shan Lei, and Hongyu Licontributed equally to this work.

*Corresponding author. E-mail: [peng.huang@szu.edu.cn](mailto:peng.huang@szu.edu.cn) (Peng Huang)

**S1 Experimental Section**

**S1.1 Synthesis of Ir SAzymes**

The Ir SAzyme was prepared following the reported protocol published elsewhere with slight modifications [S1]. Ir(acac)_3_@ZIF-8 can be prepared by the following procedure: 1.8 g Zn(NO_3_)_2_·6H_2_O and 0.3 g Ir(acac)_3_ were dissolved in 75 mL methanol at the same time; the resulting solution was then added into 20 mL of methanol containing 4.64 g 2-methyl imidazole. After vigorously stirring for 5 min, the mixed solutions were kept at room temperature without stirring for 20 h. The Ir(acac)_3_@ZIF-8 nanocrystal was collected by centrifugation, washed four times with methanol and dried in vacuum overnight. The powder of Ir(acac)_3_@ZIF-8 was then placed in a tube furnace and heated to 900 °C for 3 h at the heating rate of 5 °C min^–1^ under argon gas. The result powder was calcined twice again with the same condition as above. After calcination, the samples were cooled to room temperature to obtain Ir SAzymes.

**S1.2 Extraction of 4T1 cancer cell membrane (4T1 M)**

Briefly, The harvested 4T1 Cells were washed 3 times with PBS, and then resuspended in HM buffer (0.25 mM sucrose, 1 mM EDTA, 20 mM Hepes-NaOH, protease inhibitor, pH =7.4). Under ice bath conditions, crush with a homogenizer, centrifuge at 1500 rpm for 5 min to remove unbroken cell pellets, centrifuge the supernatant at 7500 rpm for 20 min to remove organelle pellets, and finally centrifuge at 20,000 rpm for 20 min to obtain cell membrane debris. The cell membrane fragments were extruded through 0.8 µm, 0.45 µm, and 0.22 µm filters at a time to obtain cell membrane vesicles, which were freeze-dried and stored at -80 °C for use.

**S1.3 Light to heat conversion property**

To determine the light to heat conversion activity, aqueous suspensions of Ir SAzyme with varying concentrations (0-400 μg mL^-1^) were irradiated by laser (808 nm) for 5 min at a power density of 0.8 W cm^-2^. The real-time temperature increase was recorded by an infrared thermal imager (FLIR Systems AB, Sweden). Meanwhile, the photothermal stability of Ir SAzyme (400 μg mL^-1^) was also recorded under five repetitive lasers (808 nm, 0.8 W cm^-2^) on/off cycles. The photothermal conversion efficiency (PCE) of the Ir SAzyme was calculated by the method reported by Roper *et* al. [S2]. The photothermal conversion efficiency (*η*) of IrSAzymes was calculated according to the previously reported methods, detailed calculation as following: During the photothermal heating process, the total energy balance for the system can be expressed as:

$\sum_{i} m_{i}C_{p,i}\frac{\text{d}T}{\text{d}t}=Q_{\text{NP}}+Q_{\text{sys}}-Q_{\text{diss}}$ (S1)

Where m (g) represents the mass of the solution (*m_s_*) and sample cuvette (*m_q_*), *C* (J g^-1^ ^o^C^-1^) includes the constant-pressure heat capacity of solution (*c_s_*) and sample cuvette (*c_q_*), *ΔT* (°C) is the difference between the solution temperature *T* at time *t* and the starting solution temperature *T_0_*, *Q_NP_* (mW) is determined as the energy arising from the nanoparticles, and *Q_diss_* (mW) is the thermal energy lost to the surrounding environment. In addition, *Q_S_* (mW) is the energy input by the sample cuvette and the solvent (pure DI water).

$Q_{\text{NP}}=I(1-10^{-A_{\lambda}})\eta$ (S2)

Where *I* is the laser power which is incident on the system, *A_808_* is defined as the absorbance of the IrSAzymes at the wavelength of 808 nm, and *η* is known as the photothermal conversion efficiency from the absorbed laser energy to thermal energy. Furthermore, the energy dissipation mainly occurs through the heat conduction and thermal radiation. *Q_diss_* is linear with temperature for the outgoing thermal energy, then take the form as Eq. (S3):

$Q_{\text{diss}}=hS\left(T-T_{\text{surr}}\right)$ (S3)

Where *h* (mW m^-2^·^o^C^-1^) is heat transfer coefficient, *S* (m^2^) is the surface area of the container, ΔT is the temperature change which is defined as *T*-*T_Surr_*, *T* (^o^C) is the water temperature and *T_Surr_* (^o^C) is the solution temperature ambient temperature of surrounding environment.

When the temperature rises at a maximum steady-state temperature *T_Max_* (^o^C), the system reaches the steady state. In this case, the heat input is equal to heat output, and the left side of Eq. (S1) becomes zero. We then obtain:

$Q_{\text{NP}}+Q_{\text{sys}}=Q_{\text{diss}}=hS(T_{\max}-T_{\text{surr}})$ (S4)

After removal of laser irradiation, the energy input drops to zero:

$\sum_{i} m_{i}C_{p,i}\frac{\text{d}T}{\text{d}t}=-\text{Q}_{\text{diss}}=-h\text{S}(T-T_{\text{surr}})$ (S5)

After rearrangement followed by integration:

$t=-\frac{\sum_{i} m_{i}C_{p,i}}{hS}\ln\frac{T-T_{\text{surr}}}{T_{\text{max}}-T_{\text{surr}}}$ (S6)

The time constant *τ_s_* is expressed as Eq. (S7):

$\tau_{s}=\frac{\sum_{i} m_{i}C_{p,i}}{hS}$ (S7)

A dimensionless factor *θ* is defined as Eq. (S8):

$\theta=\frac{T-T_{\text{surr}}}{T_{\text{max}}-T_{\text{surr}}}$ (S8)

Then Eq. (S9) could be expressed as:

$t=-\tau_{s}\ln\theta$ (S9):

Therefore, *τ_s_* could be calculated by linear regression of time versus negative lnθ.

And *hS* could be obtained through Eq. (S7). *Q_sys_* could be measured by replacing nanoparticles with pure solvent:

$Q_{\text{sys}}=hS(T_{\max,\text{H}_{2}\text{O}}-T_{\text{surr}})$ (S10)

At last, *η* could be calculated as:

$\eta=\frac{hS(T_{\max}-T_{\text{surr}})-Q_{\text{sys}}}{I(1-10^{-A_{\lambda}})}$ (S11)

The result of that case irradiated under 808 nm laser is that *τ_s_* = 181.4 s, obtained by linear fitting using linear cooling time and negative natural logarithm of temperature (Fig. 2c). The value of *m* and *C* are 0.2 g and 4.2 J g^-1^ ^o^C^-1^ respectively. In addition, the (T_Max_-T_Sur_) is 41.3 ^o^C, *I_808_* is 800 mW, *A_808_* is 1.91. Thus, the photothermal conversion efficiency (*η*) of Ir SAzymes at 808 nm can be calculated to be 38.1%.

**S1.4 Catalase-like activity assay of Ir SAzymes**

The CAT-like activity was analyzed the absorbance values were recorded in time scan mode at 240 nm by a spectrophotometer. Ir SAzymes: 100 μg mL^-1^, H_2_O_2_ concentration (2-50 mM). The kinetic parameters were obtained using the Lineweaver-Burk plot, 1/*V*=1/*V*_max_+*K*_m_(*V*_max_*C*), *V*: initial velocity, *V*_max_: maximum rate of conversion, *C*: substrate concentration, and *K*_m_: Michaelis constant. the change of absorption spectra.

**S1.5 POD-like activity assay of Ir SAzymes**

The kinetic analysis of Ir-SAzymess+H_2_O_2_ was performed in the presence of varying concentrations of H_2_O_2_ but at a fixed TMB concentration and vice versa. Fix the H_2_O_2_ concentration at 5 mM and vary the TMB concentration (50-1200 μM) or fix the TMB concentration at 500 μM and vary the H_2_O_2_ concentration (2-50 mM), Ir SAzymes: 10 μg mL^-1^. The optical density (OD) of oxidized TMB at 652 nm were recorded by an absorption spectrometer. The kinetic parameters were determined by the Michaelis-Menten equation: *V*=*V*_max_*C*/(*K*_m_+*C*), where *V*: initial velocity, *V*_max_: maximum rate of conversion, *C*: substrate concentration, and *K*_m_: Michaelis constant.

**S1.6 GSHOx-like activity assay of Ir SAzymes**

The consumption of GSH was monitored by microplate reader. Different concentrations of Ir SAzyms (0-400 μg mL^-1^) was mixed with GSH (5 mM) at room temperature. After incubated 4 h, 100 μL of this solution was added into 900 μL PBS, and then 4 μL 5, 5'-dithiobis-(2-nitrobenzoic acid) (DTNB) (10 mg mL^-1^) was added. After centrifugation to remove Ir SAzyme, the absorption at 412 nm of supernatant measured by microplate reader.

**S1.7 Computational method**

We have employed Vienna ab-initio simulation package (VASP) [S3, S4] to perform all the spin-polarized density functional theory (DFT) calculations within the generalized gradient approximation (GGA) using the Perdew-Burke-Ernzerhof (PBE) [S5] formulation. We have chosen the projected augmented wave (PAW) potentials [S6] to describe the ionic cores and take valence electrons into account using a plane wave basis set with a kinetic energy cutoff of 400 eV. Partial occupancies of the Kohn−Sham orbitals were allowed using the Gaussian smearing method and a width of 0.05 eV. The electronic energy was considered self-consistent when the energy change was smaller than 10^−4^ eV. A geometry optimization was done until the force was converged to 0.05 eV/Å. Grimme’s DFT-D3 methodology [S7] was used to describe the dispersion interactions among all the atoms in adsorption models of interest.

**S1.8 Evaluation of *in situ* oxygen generation**

The catalytic activity of Ir SAzymes for oxygen production was quantified using an oxygen-specific electrode integrated into a Multi-Parameter Analyzer (DZS-708, Cany). A 100 µg mL^-1^ Ir SAzymes solution, adjusted to various pH levels, was combined with a 3 wt% H_2_O_2_ solution. Oxygen generation was monitored in real-time, with measurements of the dissolved oxygen concentration (mg L^-1^) taken at 30-second intervals over a 5-minute period. Representative data were captured using CLSM.

**S1.9 Cell lines and culture conditions**

4T1 cells (Mouse breast cancer cells) and MCF-10A (human mammary cell), Human hepatocellular carcinoma cells (Huh-7), B16 cells (Mouse melanoma cells) were obtained from the Cell Bank of the Chinese Academy of Sciences (Shanghai, China). The cells were cultured in a controlled environment (37 ^o^C, 5% CO_2_) by using dulbecco's modified eagle medium (DMEM) (Gibco BRL) supplemented with 10% fetal bovine serum (FBS) and 1% penicillin/streptomycin. For normal conditions, 20% oxygen concentration was used.

**S1.10 Cellular uptake**

4T1 cells (2×10^5^ cells) were seeded in 6-well plates and incubated overnight. The cells were then incubated with 40 μg mL^−1^ RhB-labeled IGM for different time periods, after which they were analyzed using flow cytometry. For Homology targeting, different cacer cells lines (4T1/MCF-10A/Huh-7) were seeded into confocal dishes (1×10^5^ cells per well) and incubated overnight. Subsequently, the cells were incubated with 40 μg mL^−1^ RhB-labeled IGM for 4 h. Finally, the cells were observed using the ZEISS-LSM880 CLSM at different time points (𝜆_ex_=543 nm, 𝜆_em_=580 nm).

**S1.11 *In vitro* cytotoxicity**

4T1 and MCF-10A cells were seeded in 96-well plates at a density of 5×10^3^ cells per well and incubated for 24 h. After being rinsed with PBS (pH 7.4), the cells were incubated with different concentrations of IM for 24 h at 37 ºC under the same conditions. After the incubation, the relative cell viability was measured by CCK-8 assay. For *in vitro* catalytic therapy, the cells were incubated with different concentrations of IM or IGM for 4 h before being exposed to a 808 nm laser (0.8 W cm^-2^, 5 min). After irradiation, cells were incubated for another 20 h at 37 ºC, then relative cell viabilities were measured by CCK-8 assay.

**S1.12 ROS generation**

To evaluate ROS generation *in vitro,* 4T1 cells (2×10^5^ cells) were seeded in 6-well plates, incubated for 24 h, divided into same groups and followed the same procedure as intracellular oxygen estimation. The cells were carefully washed with PBS and further incubated with DCFH-DA for 15 min. Finally, the cells were analyzed via flow cytometry.

**S1.13 LPO staining**

To evaluate lipid peroxidation of the cell membrane, 4T1 cells (1×10^5^ cells) were cultured with fresh DMEM with FBS at 37 °C for 24 h, after different treatments, such cells were stained with C11-BODIPY^581/591^ dye (10×10^−6^ M) for 30 min before being observed using ZEISS-LSM880 CLSM system.

**S1.14 JC-1 assay**

4T1 cells (2×10^5^/well) were inoculated into 6-well plates and incubated overnight in an incubator. Blank medium, and medium containing IM/IGM were added to each well and incubated for 4 h. Next, the cells were irradiated by NIR-I laser (808 nm, 0.8 W cm^-2^) for 5 min and the cells were washed with PBS and digested with trypsin for 2 min, then centrifuged, and the cellular precipitate was collected. Afterwards, the cells were stained with the standard procedure of a JC-1 staining kit for 30 min, and the mitochondrial membrane potential was determined using flow cytometry.

**S1.15 Cell apoptosis and necrosis study**

To determine cellular apoptosis and necrosis, 4T1 cells were seeded in 6-well plates at a density of 2×10^5^ cells per well and incubated for 24 h. Subsequently, the culture medium was removed. The cells were incubated with fresh medium containing PBS, IM and IGM for 4 h, followed by treating with or without 808 nm laser irradiation (0.8 W cm^−2^) for 5 min, and further cultured for another 4 h. The 4T1 cells were centrifuged (1300 rpm, 3 min) and washed 2 times with binding buffer. Then the solution stained with the 250 ng mL^-1^ of annexin V-FITC and 250 ng mL^-1^ propidium iodide and incubated at 25℃ for 10 min. The 4T1 cells were centrifuged (1300 rpm, 3 min) and washed 2 times with binding buffer for flow cytometry analysis (FACS Canto Analyzer, BD Corp). The data were obtained and analyzed using CELL QUEST and FLOWJO programs.

**S1.16 Western Blotting Assay**

4T1 cells were cultured in 6-well plates and and subjected to different treatments. After washing, the cells were lysed and the proteins were extracted, and the total protein concentration of each group was determined by BCA assay. Subsequently, immunoblotting was performed using antibodies to β-actin (1:1000, Cell Signaling Technology, catalogue no. 8457S), GLUT1 (1:1000, Cell Signaling Technology, catalogue no. 12939S), HSP70 (1:1000, catalogue no. ab31010) and GPX4 (1:1000, Axil Scientific Pte Ltd., catalogue no. sc-166120), and then the desired protein level was detected.

**S1.17 Animals**

All animal studies were carried out under the protocol approved by the Animal Ethics Committee of the Center of Experiment Animals at Shenzhen University. All female BALB/c mice and BALB/c nude mice were obtained from Guangdong Medicinal Laboratory Animal Center (Guangzhou, China). Mice (4-6 weeks old) with body weights ~ 18-21 g were used in all animal studies. Distilled water and sterile food were provided to all the mice. All animals were acclimatized for 5 days prior to the treatment. To develop 4T1 tumor bearing mice model, subcutaneous injection of 4T1 cells (1×10^6^ cells per mouse) was performed into the right hind limb of nude mice.

**S1.18 *In vivo* toxicity assessment**

To assess the *in vivo* toxicity, hemolysis assay was conducted. Fresh blood was collected from the mice, diluted, and washed with PBS. Then, IGM (25, 50, 100, 200 and 400 μg mL^-1^) in PBS were mixed with the blood dispersion (0.3 mL). After incubation for 4 h at 4℃, the solutions were centrifuged at 4000 rpm for 5 min, and the absorbance of the supernatant at 541 nm was measured using a microplate reader. The hemolysis ratio of red blood cells (RBCs) is calculated by the formula as follows: hemolysis rate (%) = (A_sample_ - A_NCs_ - A_PBS_)/(A_DI water_ - A_PBS_) × 100%, where A_sample_, A_NCs_, A_PBS_, and A_DI water_ are the absorbance values of the sample, IGM solutions, PBS, and DI water, respectively.

To evaluate the biocompatibility of IGM *in vivo*, 100 μL PBS or IGM (20 mg kg^-1^) were intravenously injected into the healthy mice (n = 3), respectively. Mice were euthanized after injection for 14 days and the mice's blood was obtained for blood biochemical analysis.

**S1.19 *In vitro* and *in vivo* PA imaging**

For *in vitro* PA imaging, IGM aqueous dispersions with various concentrations (0, 25, 50, 100, 200, 400 μg mL^-1^) were filled into plastic pipes to detect their PA signal. PA imaging was conducted on a LAZR-X system (VisualSonics Inc. New York, NY) with the following parameters: Frequency: 40 MHz; 2D gain: 19 dB; PA gain: 40 dB; excitation wavelength: 808 nm.

For *in vivo* PA imaging, when the tumor size reached about 100 mm^3^, IM or IGM (10 mg kg^-1^) was intravenously injected into the 4T1 tumor-bearing mice. The photoacoustic signals of tumor tissues were acquired at various time points (0, 1, 2, 4, 8, 12, and 24 h post-injection). PA imaging was performed by a LAZR-X system (VisualSonics Inc. New York, NY) equipped with a 40 MHz, 256-element linear array transducer on tumors. Frequency: 40 MHz; 2D gain: 19 dB; PA gain: 40 dB; excitation wavelength: 808 nm.

**S1.20 *In vivo* fluorescence imaging**

To facilitate the observation of IGM in vivo distribution, a nude mouse tumor-bearing model was used for fluorescence imaging. For *in vivo* fluorescence imaging, when the tumor size reached about 100 mm^3^, IGM-IR800 (10 mg kg^-1^) was intravenously injected into the 4T1 tumor-bearing mice. The fluorescence signals of tumor tissues were acquired at various time points (0, 4, 8, 12, 24, and 48 h post-injection). In vivo fluorescence images were recorded before and after injection of IGM-IR800 at appointed times by an IVIS Spectrum system (λ_ex_ =745 nm, λ_em_ = 800 nm). After the spectra were acquired, the integrated signal intensity was calculated. Moreover, three mice were sacrificed at 8, 12, 24 and 48 h after intravenously injection. The major organs and tumor tissues collected from the sacrificed mice were observed and analyzed using IVIS Spectrum system.

**S1.21 *In vivo* treatment**

When the tumor size reached about 100 mm^3^, the tumor-bearing mice were randomly divided into the following six groups (n = 5): PBS group, Laser group, IM group, IM+laser group, IGM group, IGM+Laser group. Laser parameters: 808 nm, 0.8 W cm^−2^, 10 min. The injection dose of IM and IGM was 10 mg kg^-1^ and GOx is 0.17 mg kg^-1^ (determined by the BCA assay). For photothermal enhanced catalytic therapy, the mice were irradiated after 8 h post *i.v.* injection of PBS, IM and IGM. The [tumor](file:///E:/%25E6%2599%25AE%25E9%2580%259A%25E8%25BD%25AF%25E4%25BB%25B6/%25E6%259C%2589%25E9%2581%2593%25E8%25AF%258D%25E5%2585%25B8/Dict/8.9.4.0/resultui/html/index.html#/javascript:;) [size](file:///E:/%25E6%2599%25AE%25E9%2580%259A%25E8%25BD%25AF%25E4%25BB%25B6/%25E6%259C%2589%25E9%2581%2593%25E8%25AF%258D%25E5%2585%25B8/Dict/8.9.4.0/resultui/html/index.html#/javascript:;) and body weight of mice were monitored every 2 days for 14 days. The tumor volume was calculated by the formula: tumor volume (mm) = AB^2^/2, where A and B are the maximum length and minimum width of the tumor, respectively. The major organs (heart, liver, spleen, lung, kidney) and tumors in each group were collected for hematoxylin and eosin (H&E) staining to appraise the *in vivo* biocompatibility of IGM. And tumors of laser groups were collected after 12 h laser irradiation for H&E staining.

**S1.22 Statistical analysis**

The representative experimental data were shown as mean ± standard deviation (SD). Statistical analysis was conducted using an unpaired two-tailed Student’s t-test (*, p < 0.05; **, p < 0.01; ***, p < 0.001).

**S2 Supplementary Figures and Tables**

**
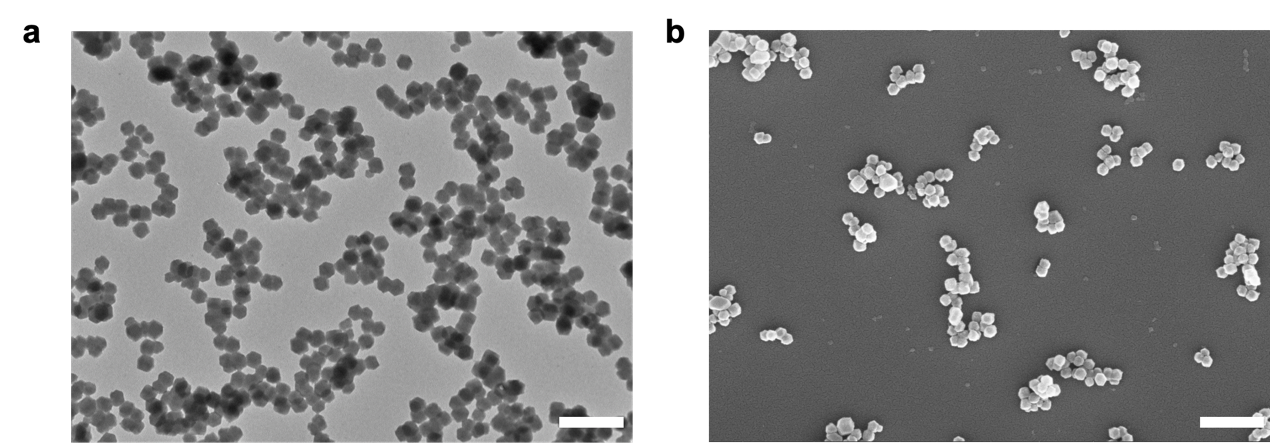
**

**Fig. S1** **a** TEM and **b** SEM image of Ir(acac)_3_-ZIF. Scale bar: 500 nm


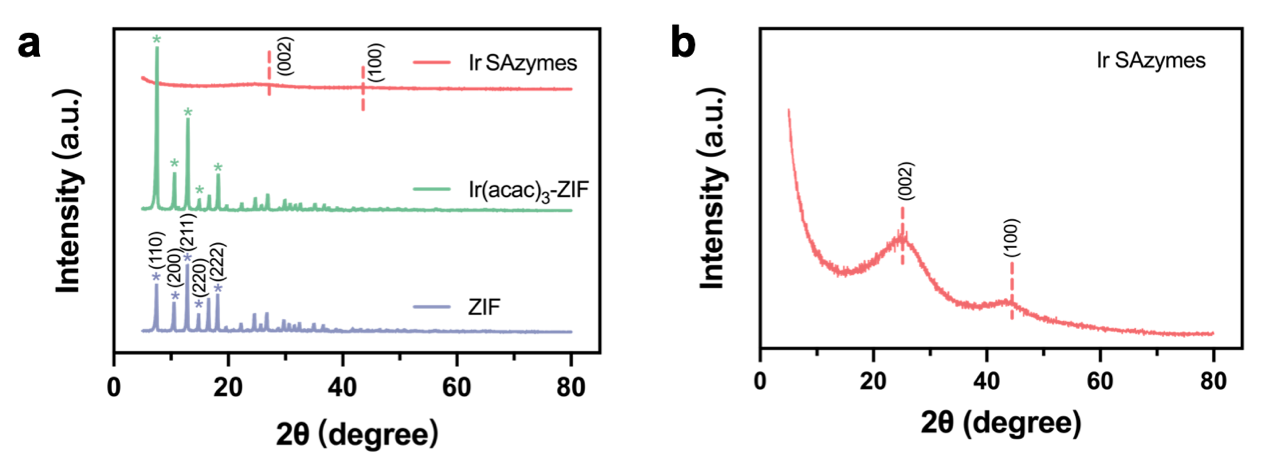


**Fig. S2 a** XRD patterns of Ir SAzymes, Ir(acac)_3_-ZIF and ZIF. **b** XRD pattern of Ir SAzymes


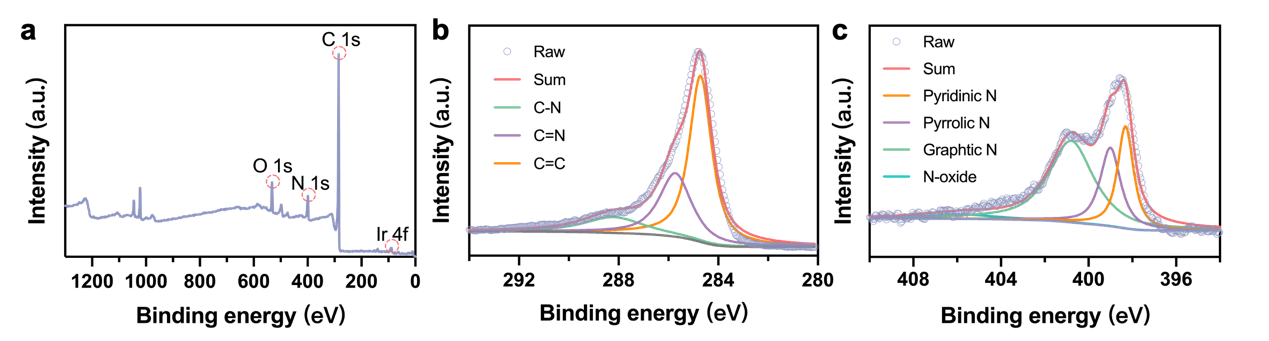


**Fig. S3 a** XPS spectrum of Ir SAzyme. **b** XPS spectra of Ir SAzyme for C 1s and **c** N 1s regions

**Table S1** EXAFS fitting parameters for various samples（*Ѕ*_0_^2^=0.819）

| Sample | Shell | *N^a^* | *R*(Å)*^b^* | *σ*^2^(Å^2^)*^c^* | Δ*E*_0_(eV)*^d^* | *R* factor |
| --- | --- | --- | --- | --- | --- | --- |
| Ir foil | Ir-Ir | 12 | 2.71 | 0.0031 | 8.3 | 0.0012 |
| IrO_2_ | Ir-O | 6.0 | 1.98 | 0.0028 | 12.7 | 0.0012 |
|  | Ir-Ir | 2.0 | 3.16 | 0.0035 |  |  |
|  | Ir-O | 8.6 | 3.45 | 0.0028 |  |  |
|  | Ir-Ir | 6.0 | 3.59 | 0.0035 |  |  |
| Ir SAzymes | Ir-N | 4.2 | 1.98 | 0.0031 | 3.7 | 0.0031 |

*^a^N*: coordination numbers; *^b^R*: bond distance; *^c^σ*^2^: Debye-Waller factors; *^d^* Δ*E*_0_: the inner potential correction. *R* factor: goodness of fit. *Ѕ*_0_^2^ was set to 0.819 for Ir, according to the experimental EXAFS fit of Ir foil by fixing coordination number as the known crystallographic value.


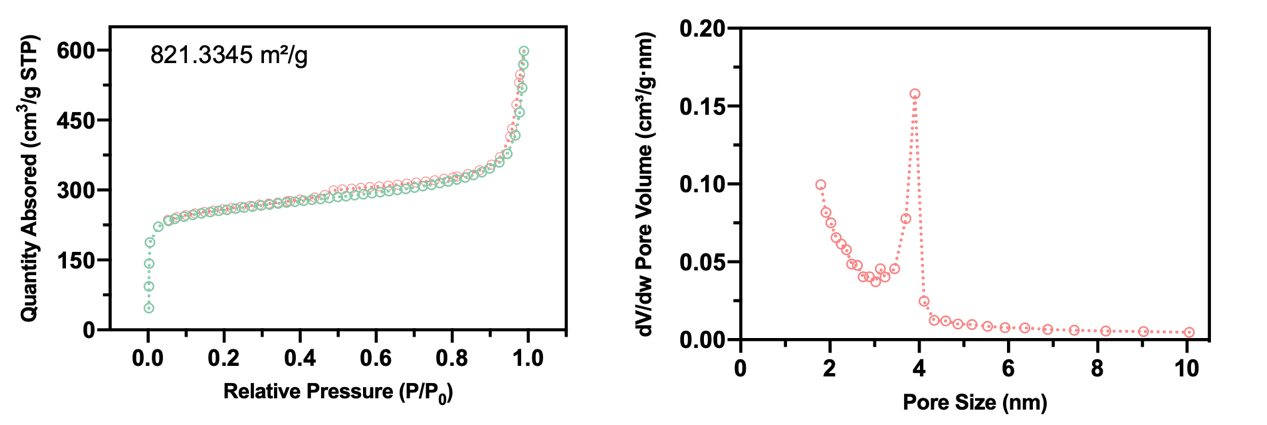


**Fig. S4** N_2_ adsorption and desorption isotherms of the Ir SAzymes


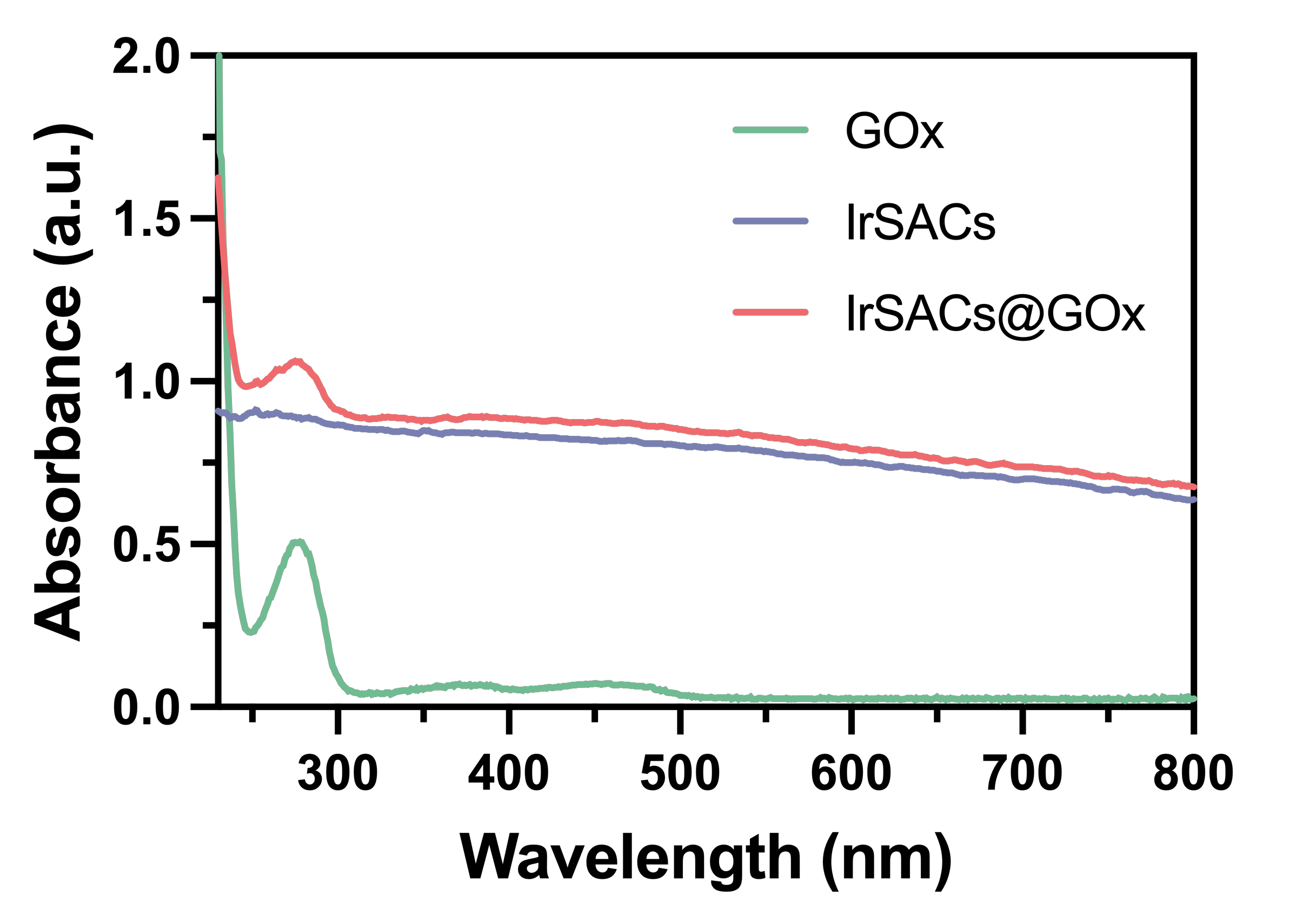


**Fig. S5** UV-Vis-NIR absorption spectra of Ir SAzyme, Ir SAzyme@GOx, and GOx

**
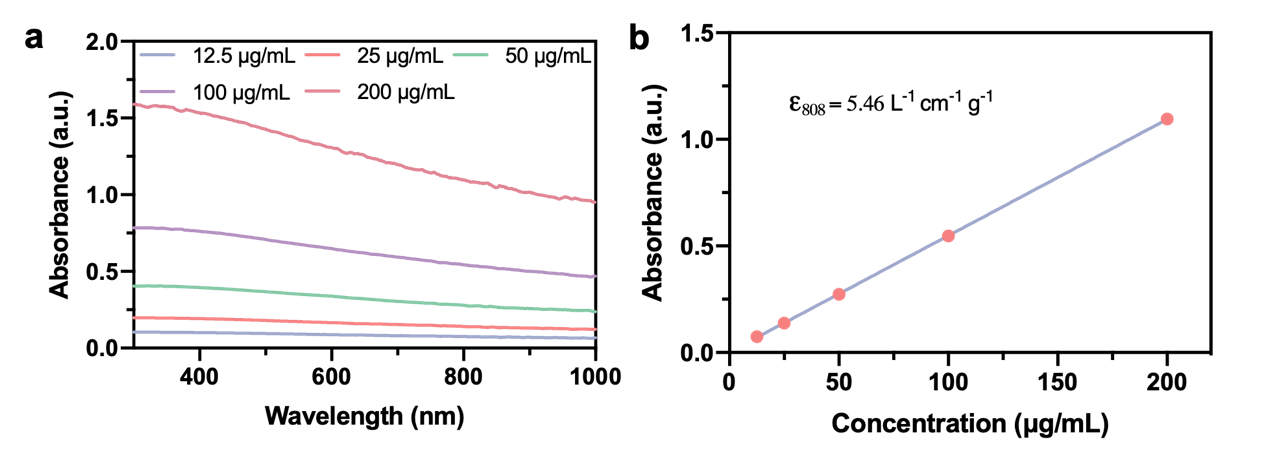
**

**Fig. S6 a** UV-Vis-NIR absorption spectra of Ir SAzymes at different concentrations. **b** The relationships between the ratio of the absorbance of Ir SAzymes at 808 nm to the length of cuvette (L) (A/L) on the IGM concentration


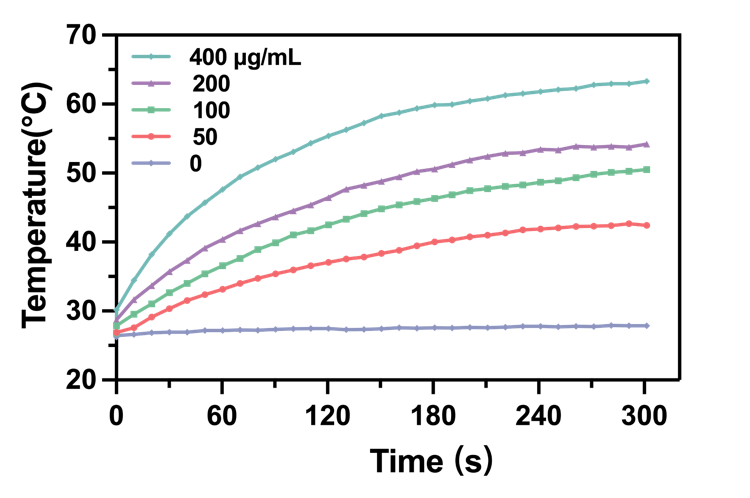


**Fig. S7** Concentration-dependent photothermal curves of Ir SAzymes (0, 50, 100, 200, 400 μg mL^-1^) under 808 nm laser irradiation for 5 min.


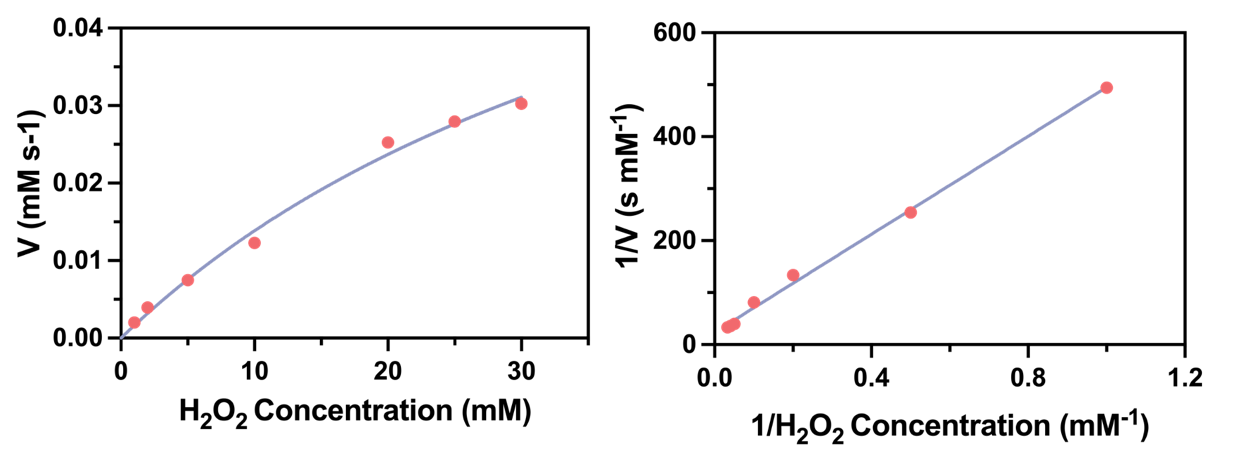


**Fig. S8 a** Kinetic analysis of Ir SAzyme with CAT-like activity. **b** Double-reciprocal plots of activities of Ir SAzyme which derived from the Michaelis equation


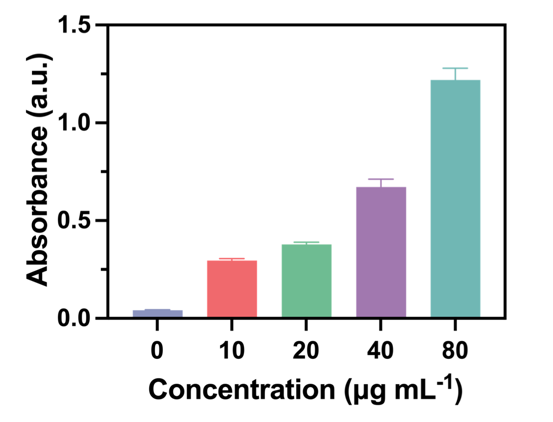


**Fig. S9** POD-like activity of Ir SAzymes by TMB chromogenic reaction


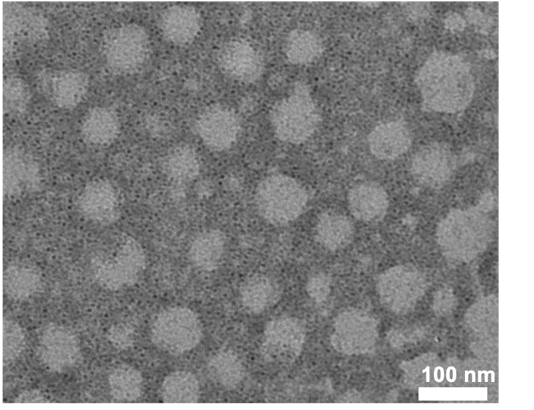


**Fig. S10** TEM image of 4T1 cell membrane vesicles

**
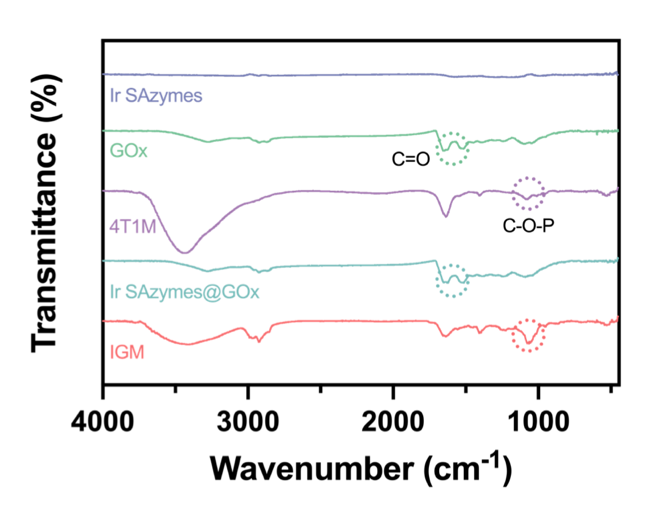
**

**Fig. S11** FTIR spectra of Ir SAzymes, GOx, 4T1M, IrSAzymes@GOx, and IGM


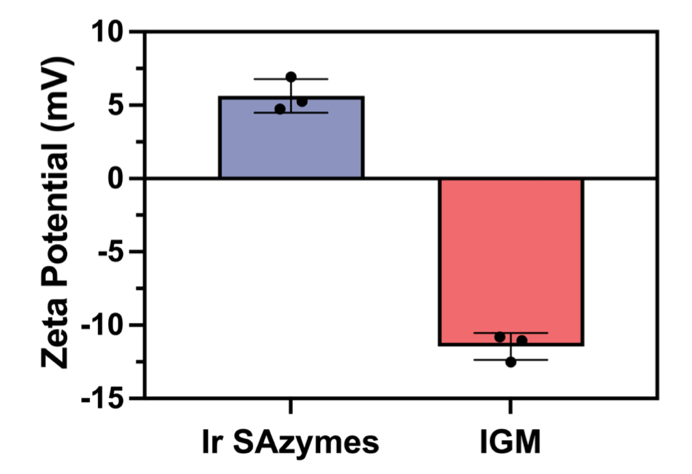


**Fig. S12** Zeta potentials of Ir SAzymes and IGM. Data are presented as mean ± SD. (n = 3)

**
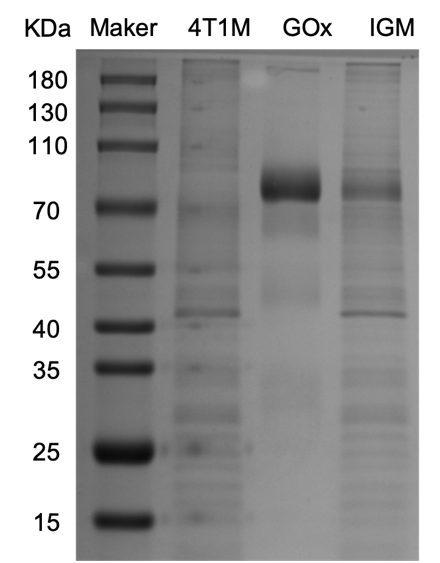
**

**Fig. S13** SDS-PAGE protein analysis


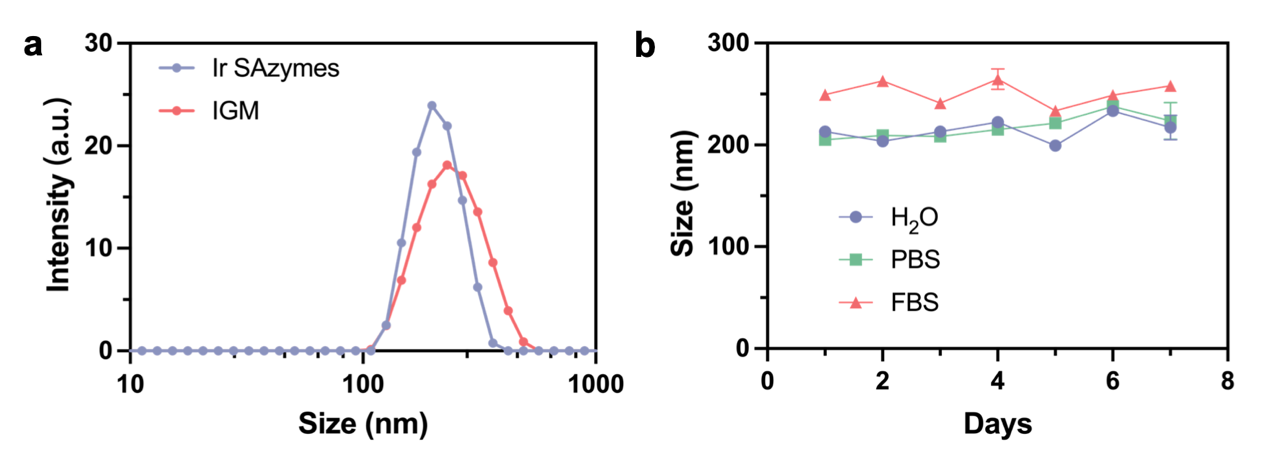


**Fig. S14** **a** Particle size determination by dynamic light scattering. **b** The hydrodynamic size profiles of IGM in different buffer solutions (H_2_O, PBS, and FBS) for 7 days


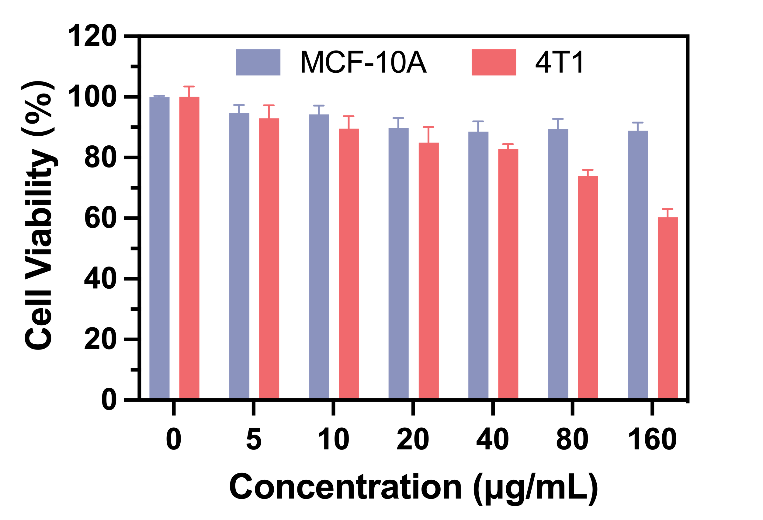


**Fig. S15** Cytotoxicity assessment of MCF-10A or 4T1 cells after treated with different concentrations of IM


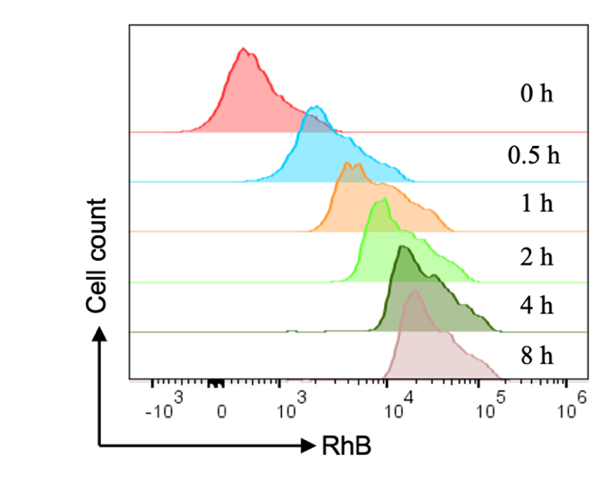


**Fig. S16** Uptake of the 4T1 cells treated with RhB labled IGM


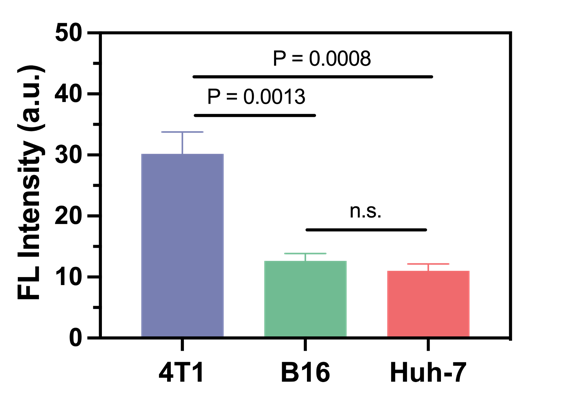


**Fug. S17** The corresponding quantification of FL intensity of various cells treated with RhB labled IGM


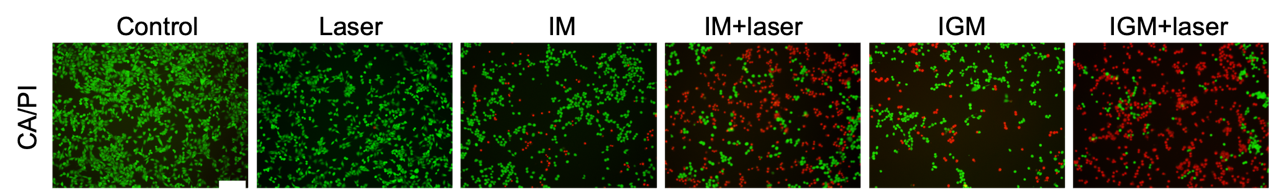


**Fig. S18** Live/dead co-stained 4T1 with GOx, IM, and IGM for 4 h with or without laser irradiation (0.8 W cm^-2^, 5 min). Scale bar is 200 μm


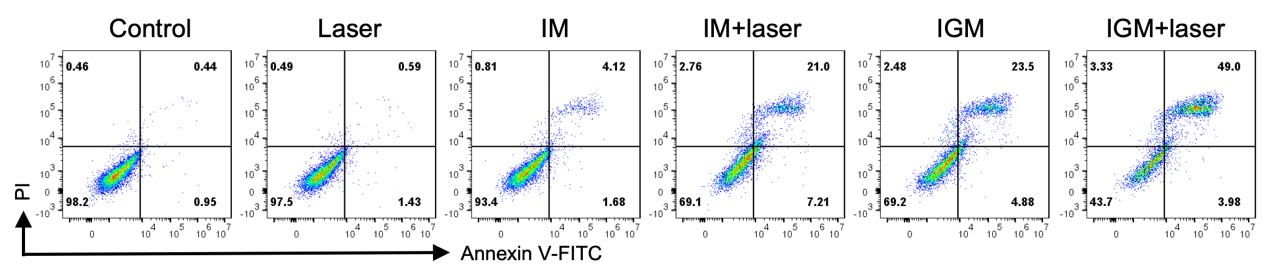


**Fig. S19** Flow cytometric measurement on cells co-stained with Annexin V-FITC and PI. The percentage of apoptotic cells was labeled in figures


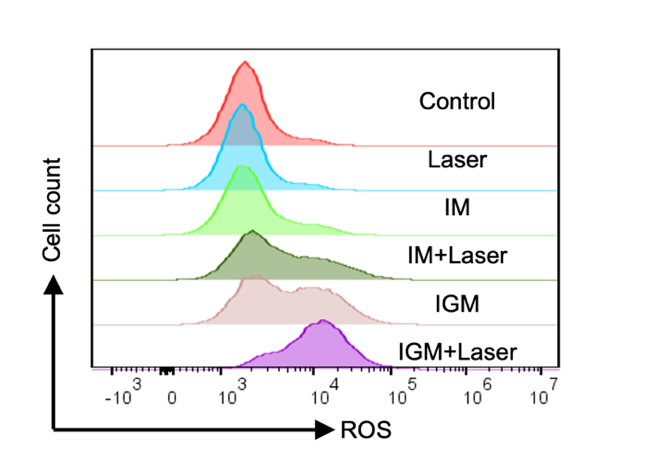


**Fig. S20** Flow cytometric analysis of the production of ROS in 4T1 cells following various treatments


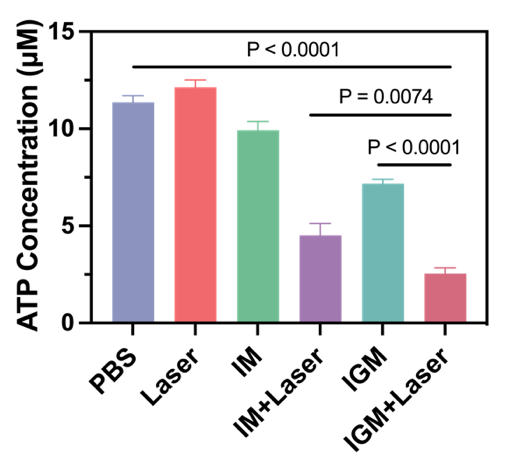


**Fig. S21** ATP assay of 4T1 cells after viaious treatments. Data are presented as mean ± SD (n = 3)


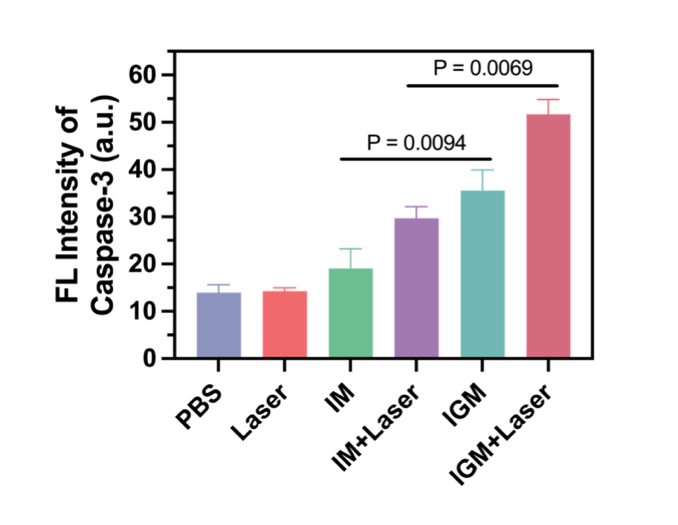


**Fig. S22** Quantification of fluorescence signals for Caspase-3 following various treatments. Data are presented as mean ± SD (n = 3)


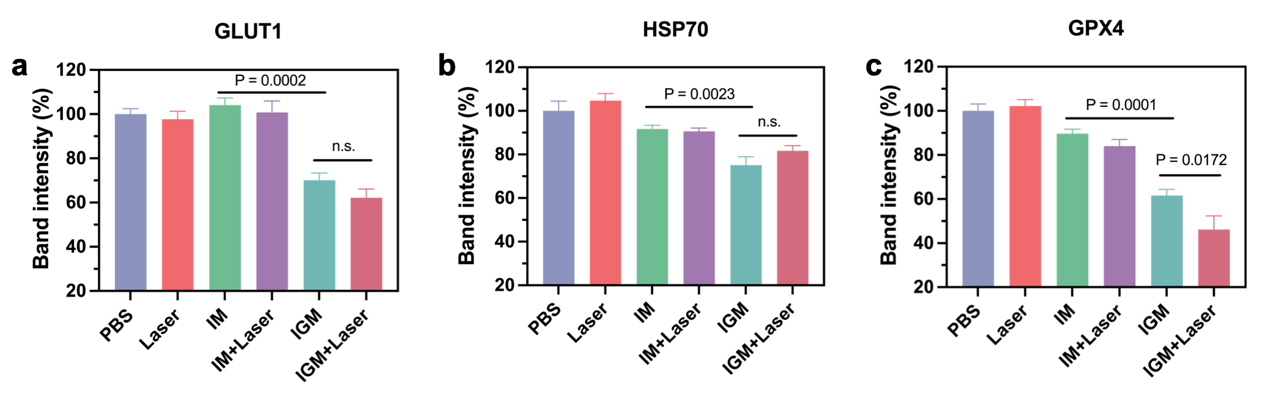


**Fig. S23** Quantitative analysis of GLUT1, HSP70 and GPX4 expression after different treatments. Data are presented as mean ± SD (n = 3)

**
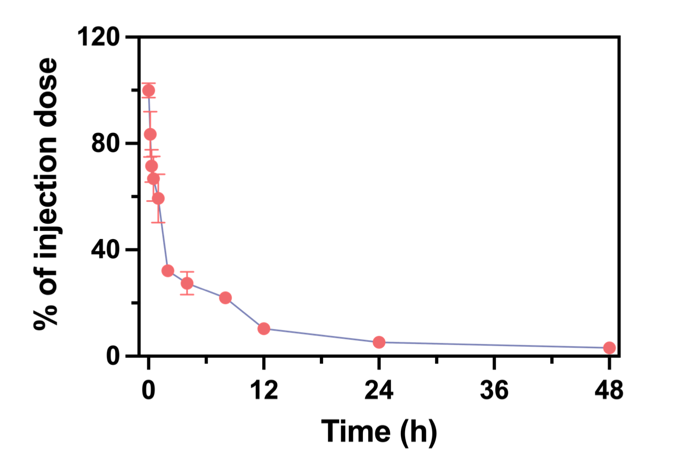
**

**Fig. S24** Plasma concentration-time curve of IR-IR800 at different time points (0, 0.17, 0.33, 0.5, 1, 2, 4, 8, 12, 24 and 48 h). Data are presented as mean ± SD (n = 3)

**Table S2** Pharmacokinetic parameters of IGM-IR800 Parameters

| Parameters | No.1 | No.2 | No.3 | Mean | SD |
| --- | --- | --- | --- | --- | --- |
| AUC_(0-∞)_^a^ | 277.42 | 281.634 | 256.562 | 271.872 | 13.425 |
| MRT_(0-∞)_^b^ | 7.978 | 7.28 | 5.503 | 6.92 | 1.276 |
| t_1/2_^c^ | 5.356 | 4.675 | 2.379 | 4.137 | 1.56 |

a: plasma concentration-time curve (mg/L*h). b: mean residence time (h). c: half life (h).

**
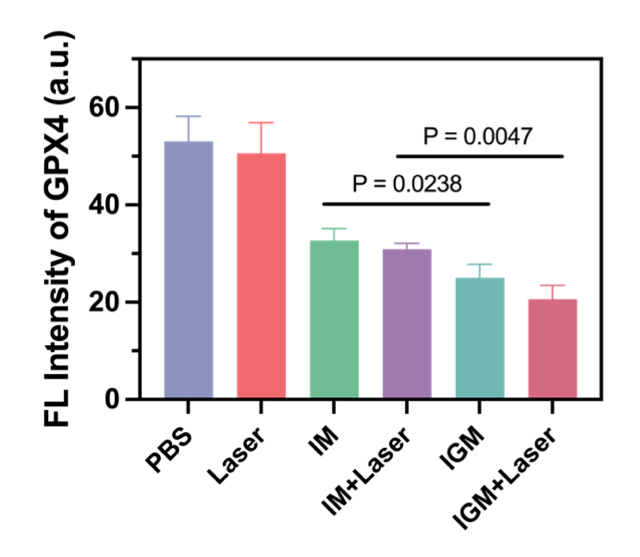
**

**Fig. S25** Quantitative analysis of GPX4 FL intensity in 4T1 tumor slices after different treatments. Data are presented as mean ± SD (n = 3)


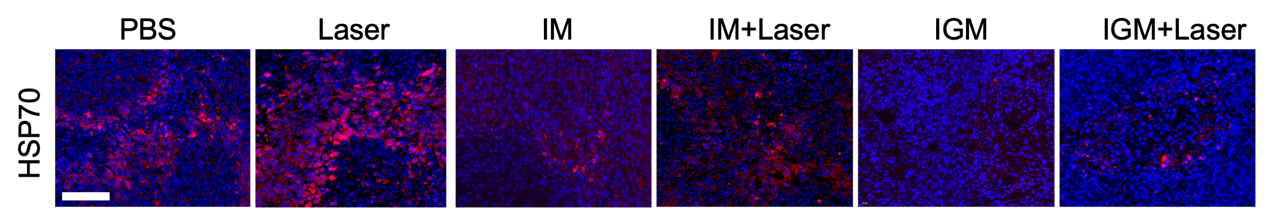


**Fig. S26** HSP70 staining images of tumor sections after different treatments. Scale bar: 100 μm. Data are presented as mean ± SD (n = 3)


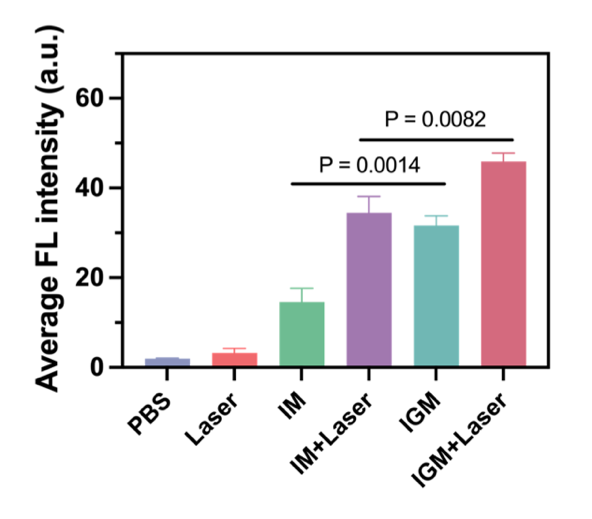


**Fig. S27** Quantification of the positive rates of TUNEL staining images of tumor tissues after indicated treatments. Data are presented as mean ± SD (n = 3)


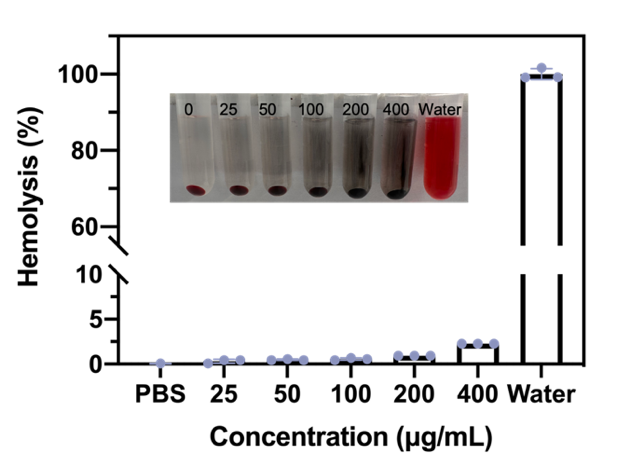


**Fig. S28** Hemolysis of IGM solution at various concentrations. Data are presented as mean ± SD (n = 3)

**
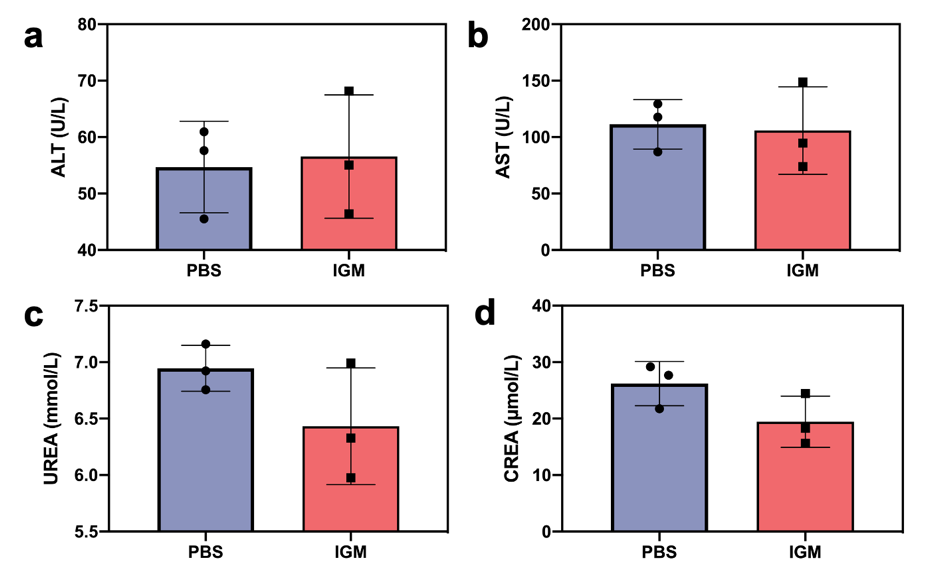
**

**Fig. S29** Blood biochemistry analysis (ALT, AST, UREA, and CREA) of healthymice after intravenously injected with saline or IGM for 14 days. Data are presented as mean ± SD. (n = 3)


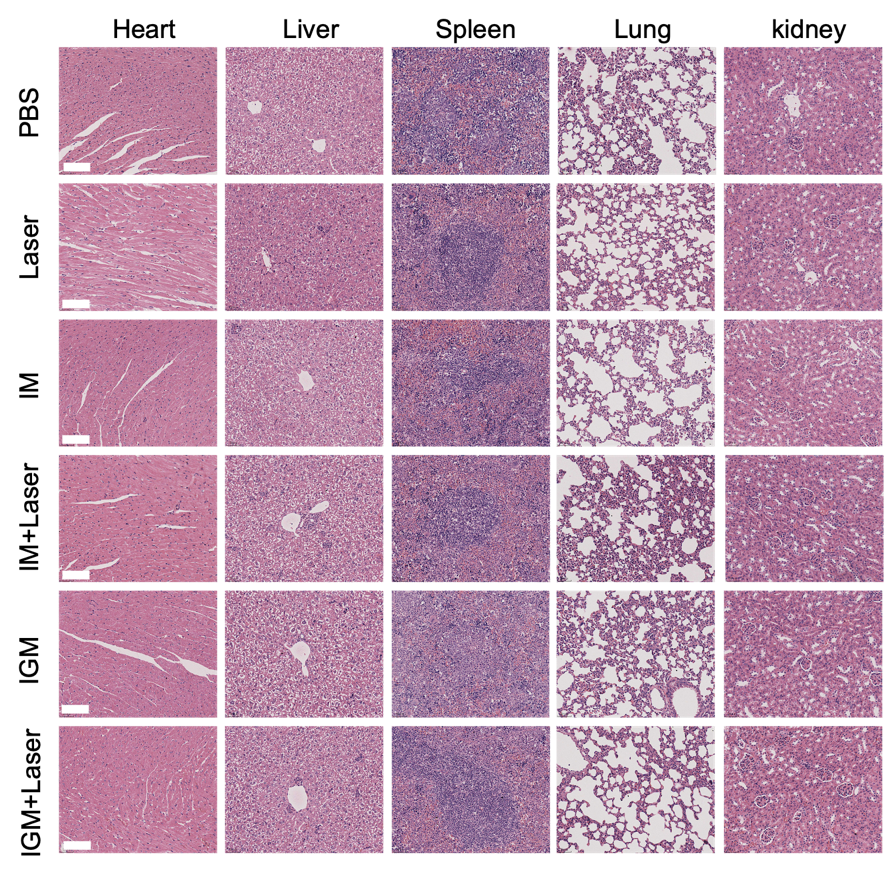


**Fig. S30** H&E staining of major organs collected from different groups after indicated treatments. Scale bar is 100 μm

**Supplementary References**

1. Z. Li, Y.J. Chen, S.F. Ji, Y. Tang, W.X. Chen et al., Iridium single-atom cas on nitrogen-doped carbon for formic acid oxidation synthesized using a general host-guest strategy. Nat. Chem. **12**, 764-772 (2020). <https://doi.org/10.1038/s41557-020-0473-9>
2. D.K. Roper, W. Ahn, M. Hoepfner, Microscale heat transfer transduced by surface plasmon resonant gold nanoparticles. J. Phys. Chem. C **111**, 3636-3641 (2007). <https://doi.org/10.1021/jp064341w>
3. G. Kresse, J. Furthmuller, Efficiency of ab-initio total energy calculations for metals and semiconductors using a plane-wave basis set. Comput. Mater. Sci. **6**, 15-50 (1996). <https://doi.org/10.1016/0927-0256(96)00008-0>
4. G. Kresse, J. Furthmuller. Efficient iterative schemes for ab initio total-energy calculations using a plane-wave basis set. Phys. Rev. B **54**, 11169-11186 (1996). <https://doi.org/10.1103/PhysRevB.54.11169>
5. J.P. Perdew, K. Burke, M. Ernzerhof, Generalized gradient approximation made simple. Phys. Rev. Lett. **77**, 3865-3868 (1996). <https://doi.org/10.1103/PhysRevLett.78.1396>
6. G. Kresse, D. Joubert, From ultrasoft pseudopotentials to the projector augmented-wave method. Phys. Rev. B. **59**, 1758-1775 (1999). <https://doi.org/10.1103/PhysRevB.59.1758>
7. S. Grimme, J. Antony, S. Ehrlich, H. Krieg, A consistent and accurate ab initio parametrization of density functional dispersion correction (DFT-D) for the 94 elements H-Pu. J. Chem. Phys. **132**, 154104 (2010). <https://doi.org/10.1063/1.3382344>
